# The effects of consumption of interesterified fats rich in palmitic acid compared with stearic acid on intermediary markers of cardiometabolic disease risk: a randomized controlled trial in healthy adults

**DOI:** 10.1016/j.ajcnut.2025.09.025

**Published:** 2025-09-18

**Authors:** Wendy L Hall, Eleanor Wood, Peter J Joris, Harry A Smith, Alice Creedon, Tyler Maher, Johanna H Bruce, Ronald P Mensink, Sarah E Berry

**Affiliations:** 1 Department of Nutritional Sciences, School of Life Course and Population Sciences, King’s College London, United Kingdom; 2 Department of Nutrition and Movement Sciences, NUTRIM Institute of Nutrition and Translational Research in Metabolism, Maastricht University, Maastricht, The Netherlands; 3 Centre for Nutrition, Exercise, and Metabolism, Department for Health, University of Bath, Bath, United Kingdom; 4 ADM UK Ltd, Erith, United Kingdom

**Keywords:** interesterified fats, palmitic acid, stearic acid, dietary fats, lipids, endothelial function, inflammation, cardiovascular risk factors, randomized controlled trial, postprandial metabolism

## Abstract

**Background:**

Interesterified (IE) fats rich in palmitic acid (16:0) and stearic acid (18:0) are widely used hard fats. Comparative effects of commercially relevant palmitic or stearic acid-rich IE fat blends on cardiometabolic disease (CMD) risk markers remain unclear. It was hypothesized that functionally matched palmitic and stearic acid-rich fats would have equivalent effects on plasma total:high-density lipoprotein (HDL) cholesterol ratio within a predefined equivalence margin of ±0.09.

**Objectives:**

This study aimed to compare the effects of diets enriched in palmitic or stearic acid, delivered as commercially relevant IE fats and incorporated at realistic levels of dietary intake, on plasma lipid concentrations and other CMD risk markers in healthy participants.

**Methods:**

In a randomized, crossover trial at 2 centers, 51 healthy participants (aged 35–65 y) consumed diets with either palmitic acid-rich or stearic acid-rich IE fats (10% energy intake) for 6 wk per arm, with a 4-wk washout. IE fats were incorporated into muffins and margarine spreads. The primary outcome was total:HDL cholesterol ratio; secondary outcomes included HDL and low-density lipoprotein cholesterol and fasting and postprandial triacylglycerol, lipoproteins, apolipoproteins, insulin sensitivity, vascular endothelial function, inflammation markers, and fasting liver fat (via proton magnetic resonance spectroscopy, subgroup). Linear mixed models were used to assess intervention effects.

**Results:**

Forty-seven participants (51% female, mean age 52 ± 8 y; body mass index 25.2 ± 2.8 kg/m^2^) completed the study. No significant differences were observed in total:HDL cholesterol ratio between interventions (stearic compared with palmitic mean difference: 0.05; 95% confidence interval [CI]: −0.08, 0.18). Interleukin-10 concentration decreased after consuming palmitic relative to stearic acid-rich IE fats (−0.14 ng/L; 95% CI: −0.23, −0.06 ng/L). No effects were observed on other outcomes.

**Conclusions:**

Commercially relevant palmitic acid-rich IE fats consumed at 10% energy for a 6-wk period do not adversely affect total:HDL cholesterol ratio or other CMD risk markers compared with stearic acid-rich fats. Long-term effects could not be determined from this trial.

This trial was registered at clinicaltrials.gov as NCT04418102.

## Introduction

Random interesterification (IE) is extensively used by the food industry to create fats with desirable functional characteristics for applications such as spreads and bakery products. The process produces fats with minimal *trans* fatty acids and with lower SFA content. IE involves chemically or enzymatically rearranging fatty acids on the glycerol backbone of triacylglycerols (TAGs), modifying the melting point and crystalline structure without altering the fatty acid composition. These structural changes may affect digestion, absorption, and metabolism, with potential implications for cardiometabolic health.

Although the relationships between dietary fatty acids, blood lipids, and cardiovascular disease (CVD) risk are well established, extrapolating the health effects of IE fats from their non-IE counterparts may be misleading. For example, stearic acid (18:0) is considered lipid neutral, whereas palmitic acid (16:0) raises blood cholesterol. However, these effects may differ between IE forms because of changes in fat structure and metabolism. IE fats rich in either palmitic or stearic acid are commonly used in bakery, confectionery, creams, and margarine spreads for their structural functionality, shelf life, and mouthfeel. In Europe, IE blends of palm stearin (palmitic acid-rich) and smaller amounts of lauric acid (12:0)-rich palm kernel oil or coconut oil are common, whereas in North America, stearic acid-rich blends from fully hydrogenated soybean oil predominate. Despite widespread use, the relative health effects of these IE fats, when consumed at realistic dietary levels, remain uncertain.

Most studies on dietary fats focus on fasting lipids or single postprandial meals, which may not capture the cumulative effects of IE fat intake. Postprandial lipemia, an independent CVD risk factor [1], is influenced by background diet [2,3]. Research from our group and others suggests that IE of palm oil or palm olein reduces postprandial lipemic responses [[4], [5], [6], [7]] and alters gut hormone release without affecting glucose and insulin concentrations [8]. Chronic studies on palmitic acid-rich IE fats [[9], [10], [11], [12], [13]] generally show effects on lipids or insulin sensitivity, but many use fats or intake levels lacking commercial relevance. Given the growing use of IE fats, studies using commercially relevant and functionally equivalent palmitic compared with stearic acid-rich IE fats at realistic intake levels are needed.

The primary aim of this study was to compare the effects of realistic chronic intake of the most consumed IE fat blends—either palmitic acid-rich or stearic acid-rich—on intermediary cardiometabolic disease (CMD) risk markers, with total:HDL cholesterol ratio as the primary outcome. This ratio integrates atherogenic and protective lipoprotein effects and was widely regarded at the time of study design (2018–2019) as a stronger predictor of CVD risk than individual lipids [14]. More recently, findings from the Atherosclerosis Risk in Communities (ARIC) study demonstrated that the total:HDL cholesterol ratio discriminated CVD risk even at lower LDL cholesterol and non-HDL cholesterol concentrations [15], further supporting its relevance as a clinical outcome. A secondary aim was to assess postprandial responses before and after chronic exposure. We hypothesized that palmitic acid-rich IE fat would have equivalent effects to a functionally matched stearic acid-rich IE fat on total:HDL cholesterol ratio, within a predefined equivalence margin of ±0.09.

## Methods

Ethical approval for the InterSat study was obtained from King’s College London (KCL) Research Ethics Committee and Medical Ethical Review Committee of Maastricht University (MU) and the university hospital (METC azM/UM). The study was registered at clinicaltrials.gov as NCT04418102 and conducted in accordance with the ethical standards of the 2013 Declaration of Helsinki. All participants provided written informed consent. Recruitment began in February 2020 and was completed in March 2022. The full intervention phase (including both dietary arms and washout) was completed by all participants in August 2022. There was no additional follow-up beyond the end of the second intervention period.

### Study locations and population

This study was conducted at 2 centers: KCL (Department of Nutritional Sciences, King’s College London, United Kingdom) and MU (Department of Nutrition and Movement Sciences, Maastricht University, The Netherlands). Participants were enrolled by study investigators (PJJ and EW). The inclusion criteria for the study required participants to be apparently healthy males and females, aged 35-65 y (reflecting the age range when total:HDL cholesterol ratio is more likely to be raised but when statin prescription is less common and in accordance with our previous studies of a similar design [16]), nonsmokers, with accessible veins for blood sampling. Participants needed to be willing to comply with the study protocol, have a general practitioner, agree to receive information about medically relevant test results, and provide signed informed consent. For the MU participants, there was an additional requirement of no contraindications for magnetic resonance spectroscopy procedures.

Exclusion criteria included having medical conditions or a history that could impact study measurements (e.g., myocardial infarction, angina, thrombosis, stroke, cancer, liver or bowel disease, or diabetes), use of plant sterol/stanol-enriched foods or supplements within 3 mo before screening, BMI outside the range of 20 to 35 kg/m^2^, plasma total cholesterol ≥7.5 mmol/L, TAG ≥3 mmol/L, glucose ≥7 mmol/L, or abnormal full blood count or liver function results. Individuals with blood pressure (BP) ≥140/90 mm Hg, current use of antihypertensive or lipid-lowering medications, alcohol intake ≥21 units per week, current smokers (or those who quit within the last 6 mo), or those with a ≥20% 10-y CVD risk (QRISK3 score) were excluded. Additionally, exclusions applied to active blood donors, those using oral antibiotics (except topical) in the past 40 d, individuals reporting significant weight changes (≥3 kg in 2 mo), medically prescribed diets, allergies/intolerances to test products, participation in other nutritional or biomedical trials within 3 mo, and night shift workers (midnight to 06:00) within 2 wk prior to or during the study.

### Study design

**FIGURE 1:**
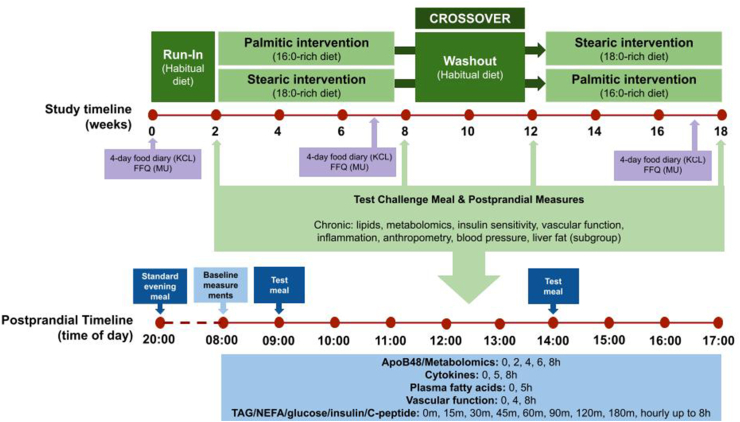
Overall InterSat study design. Abbreviations: FFQ, food frequency questionnaire; KCL, King’s College London; MU, Maastricht University; NEFA, non-esterified fatty acids; TAG, triacylglycerol.

A randomized, double-blind, crossover design study (Figure 1) was conducted to investigate chronic and acute effects of palmitic acid-rich compared with stearic acid-rich IE fats. Participants were randomly assigned to dietary intervention codes using computer software by research staff at each center after confirmation of eligibility at the screening visit. Study foods were labeled with dietary intervention codes, and test fat allocation was blinded to both participants and investigators involved in data collection and analysis. After a two-week run-in period following habitual diets (Figure 1) to familiarize participants with study procedures, chronic effects of test fats were investigated in a six-week intervention arm, with a minimum four-week washout before crossing over to the other intervention arm. Postprandial effects were tested over an eight hour period at weeks 0 and 6 of each intervention arm.

Participants were asked to complete a 4-d food diary (at KCL) or food frequency questionnaire (FFQ; at MU) prior to the initial visit; this was repeated on week 5 of each IE intervention period. The FFQ has previously been found to correlate with 3-d food records (unpublished results) and erythrocyte linoleic acid concentrations [17]. To standardize background diet during the 24 h before study visits, participants consumed an identical low-fat (<30 g) evening meal on each occasion, avoided nitrate-rich foods, and avoided exercise and alcohol. Participants were asked to fast for 12 h before each visit, only consuming low-nitrate bottled water (to avoid background variability in nitric oxide production). Participants were asked to travel to the laboratory via public or private transport (i.e., not by bike or foot). Participants provided all measurements at the research center. They also collected fortnightly supplies of IE muffins and spread (detailed in the next section) at the baseline visit of each arm.

#### Subgroups and site-specific measurements

Identical protocols were followed at the two study centers (KCL and MU), except as noted here: A subgroup at each center underwent postprandial testing at weeks 0 and 6 of each intervention arm. Fasting liver fat was measured only at MU. Endothelial function (fasting and postprandial) was assessed in all participants at KCL and in the postprandial testing subgroup only at MU.

### Dietary intervention

The dietary intervention comprised test foods (spreads and muffins, identical in appearance and taste) containing palmitic acid-rich and stearic acid-rich IE hardstocks (Table 1), referred to hereafter as the Palmitic and Stearic interventions, respectively. In the fats and oils industry, “hardstocks” refer to hard fats that are blended with other oils and fats to create fat blends suitable for various applications, such as pastries, biscuits, creams, and confectionery. Test fats were selected to represent palmitic acid-rich and stearic acid-rich IE hardstocks commonly used for margarine spread products and formulated to be matched for functionality. Following extensive research of IE fats used commercially, in collaboration with industry partners (ADM (UK) Ltd), we identified that the most consumed palmitic acid-rich IE fat was a blend of palm stearin/kernel hardstock (∼50% palmitic acid; solid fat content 60% at 20°C and 22% at 35°C); this palmitic acid-rich hardstock, when combined with rapeseed oil at ∼60:40 ratio, is commonly used in the manufacture of margarine and spread products in the United Kingdom, and so this comprised the formulation of the Palmitic spread. An alternative formulation rich in stearic acid was developed to closely replicate the functional properties of the palmitic acid hardstock, particularly firmness during spreading and melt-in-the-mouth characteristics. This was achieved by matching the solid fat content at refrigeration temperature (10°C) and oral temperature (35°C), ensuring comparable performance in real food applications. This functionally equivalent stearic acid-rich IE hardstock consisted of a blend of fully hydrogenated rapeseed oil/coconut oil/high oleic sunflower oil/sunflower oil (∼40% stearic acid; solid fat content 59% at 20°C and 20% at 35°C), which was combined with rapeseed oil at a ∼60:40 ratio to yield the Stearic spread.

**TABLE 1:** Fatty acid composition of hardstock test fats (mol%).

Fatty acid	Stearic	Palmitic
C6:0, caproic acid	0.2	0.0
C8:0, caprylic acid	3.0	1.5
C10:0, capric acid	2.1	2.1
C12:0, lauric acid	16.2	9.1
C14:0, myristic acid	6.3	3.9
C16:0, palmitic acid	7.0	48.8
C17:0, margaric acid	0.1	0.1
C18:0, stearic acid	40.5	5.0
C18:1-c, oleic acid	17.7	23.9
C18:1-t, elaidic acid	0.1	0.1
C18:2-c, linoleic acid	4.8	5.1
C18:2-t, linoelaidic acid	0.2	0.2
C18:3-c, α-linolenic acid	0.1	0.0
C20:0, arachidic acid	1.0	0.3
C20:1-c, gadoleic acid	0.1	0.1
C22:0, beheric acid	0.5	0.1
Total	99.9	100.3

Hardstock test fats were 16:0-rich (palmitic) and 18:0-rich (stearic) interesterified (IE) fats. The palmitic acid-rich IE fat consisted of a blend of palm stearin/kernel hardstock (∼50% palmitic acid; solid fat content 60% at 20°C and 22% at 35°C). The stearic acid-rich IE fat consisted of a blend of fully hydrogenated rapeseed oil/coconut oil/high oleic sunflower oil/sunflower oil (∼40% stearic acid; solid fat content 59% at 20°C and 20% at 35°C).

The dietary intervention was designed to provide 10% of daily energy requirements as hardstock test fats as this proportion can be substituted for other foods using formulated test foods without affecting total energy and fat intakes. This level of substitution equated to an additional 5% energy from palmitic acid or 4% from stearic acid. In adults aged >20 y, current median intake estimates [18] of palmitic and stearic acids are 6.5% and 3% energy, respectively. Therefore, it is estimated that the additional palmitic and stearic acids provided by test fats, which would also be likely to displace some palmitic and stearic acids in the background diet, will fall within the 95th percentile of intakes.

Participants were allocated to energy requirement categories, estimated by the Henry equation [19] to establish basal metabolic rate (BMR). BMR was multiplied by a physical activity level multiplier assuming a sedentary lifestyle (1.2) to estimate minimum daily energy requirements. Energy requirement brackets of 200 kcal (837 kJ) increments were implemented for this purpose, facilitating calculation of the target number of muffins (∼80 kcal/335 kJ per muffin) and the amount of spread needed to achieve 10% of total daily energy intake from IE hardstocks. Each muffin contained 3.3 g hardstock (test fat). For example, an individual whose estimated daily total energy expenditure was 1700 kcal (7113 kJ) consumed 18.9 g/170 kcal (711 kJ) per day of hardstock, 9 g from spread, and 9.9 g from muffins. Spreads contained either *1*) Palmitic intervention: 54% IE palm stearin/kernel hardstock (palmitic acid, 49%; stearic acid, 5%) blended with 36% rapeseed oil (final spread: palmitic acid, 32%; stearic acid, 4%); or *2*) Stearic intervention: 54% IE fully hydrogenated rapeseed oil/coconut oil/high oleic sunflower oil/sunflower oil hardstock (palmitic acid, 7%; stearic acid, 41%) blended with 36% rapeseed oil (final spread: palmitic acid, 6%; stearic acid, 25%). Muffins were supplied in different flavors according to participant preferences: savory (garlic and parsley, chili, cheese) and sweet (lemon, banana, orange). The ingredients, energy, and macronutrient composition of the muffins per 100 g are provided in Supplemental Table 1.

Anthropometric measures were measured every 2 wk. In case of weight gain/loss (that was >2 kg), the nutritionist adjusted the supply of experimental products accordingly.

#### Compliance

A range of approaches was used to evaluate and encourage compliance with interventions, including 3-d food records, study diaries, and requests for returns of empty packaging of consumed study foods. Additionally, changes in fasting plasma fatty acids (namely palmitic and stearic acids) were used as an objective indicator of compliance.

### Postprandial test meal challenge

The postprandial subgroup remained in the research center after the fasted measurements, whereupon they underwent a 2-meal postprandial test meal challenge (Supplemental Table 1), as described previously [20]. Before consumption of any food or drink, a cannula was inserted into a vein in the arm, and blood was collected followed by an endothelial function measurement. Participants then consumed the first test meal, and additional measurements were made at regular intervals for ≤8 h postprandially as outlined in Figure 1.

The first meal provided 50 g test fat (the same test fat as the chronic dietary intervention allocated for that arm), 85 g carbohydrate, and 15 g protein delivered as a muffin and a milkshake. A second test meal (cheese muffins) was given at 5 h (containing 30 g of the same test fat) to provide an additional challenge and mimic a real-life scenario of sequential meals.

### Outcome variables

#### Primary outcome

The study was designed to detect noninferiority of the interventions and was therefore powered on equivalence for total:HDL cholesterol ratio to demonstrate that there are no clinically significant differences between interventions. An equivalence margin in total:HDL cholesterol ratio of ±0.09 was assumed based on data from the Prospective Studies Collaboration [14]. A difference in ratios of <0.09 equates to a <2% difference in CVD risk.

#### Secondary outcomes

These included fasting and postprandial lipids (non-HDL cholesterol, LDL cholesterol, HDL cholesterol, lipoprotein average diameter, lipoprotein subclass particle concentration, lipoprotein (a) [Lp(a)], Apolipoprotein [Apo]A1 and ApoB48, ApoB100, total ApoB, and TAG); endothelial function, assessed by flow-mediated dilatation (FMD) of the brachial artery using ultrasound, an important noninvasive indicator of large-artery endothelial function (in a subset at MU) [21,22]; BP; serum cytokines (IL-6, IL-8, IL-10, vascular endothelial growth factor [VEGF], TNF-α, IL-1α, IL-1β, monocyte chemoattractant protein-1 [MCP-1], and epidermal growth factor [EGF]) and serum glycoprotein acetyls (GlycA; (nuclear magnetic resonance [NMR] biomarker reflecting systemic inflammation via glycosylated acute phase proteins); and markers of glucose homeostasis (glucose, insulin, HOMA-IR, C-peptide, and nonesterified fatty acid [NEFA] concentrations). Body weight and waist circumference were measured on each test day. At MU only, fasting liver fat was measured.

### Biochemical analysis

Blood samples were collected and centrifuged (1700 rpm, 10 min, 4°C) to separate serum or plasma and then stored at −80°C. Serum/plasma samples from both research centers were analyzed together. Biochemical assays were performed using a Siemens ADVIA 1800 autoanalyzer for serum lipid profiles (without replication), a Centaur XP autoanalyzer for insulin and C-peptide (without replication), and manually at Affinity Biomarker Labs using ELISA kits for ApoB48 and ApoB100 (in duplicate). Samples were handled per manufacturer instructions, avoiding repeated freeze-thaw cycles. Standard calibration curves ensured accurate quantification. Siemens Healthineers reagents were utilized for total cholesterol (CHOL-2, catalog number 10376501; linear range: 0.26–17.48 mmol/L), HDL-cholesterol (Direct HDL, catalog number 06521559; linear range: 0.10–3.0 mmol/L), LDL-cholesterol (Direct LDL, catalog number 09796248; linear range: 0.09–25.9 mmol/L), triglycerides (TRIG-2, catalog number 10335892; linear range: 0.11–6.22 mmol/L), and Lp(a) (LPA, catalog number 10361942; detection limit: 100 mg/L, upper limit: 850 mg/L). Siemens Healthcare Diagnostics reagents were used to determine insulin (catalog number 2230141; detection limit: 0.5 mIU/L, upper limit: 300 mIU/L) and C-peptide (catalog number 03649928; detection limit: 0.05 μg/L, upper limit: 30 μg/L) concentrations via 2-site sandwich immunoassays with chemiluminometric detection. ApoB48 (Human Apo B-48 ELISA kit, catalog number 637-10641, Shibayagi Co., Ltd.; detection limit: 2.5 μg/L, upper limit: 160 μg/L) and ApoB100 (Human Apo B-100 ELISA kit, catalog number 27181, IBL, distributed by Tecan Group Ltd.; detection limit: 0.03 mg/L, upper limit: 8.4 mg/L) concentrations were quantified in duplicate using sandwich ELISA with absorbance measured at 450 nm on a Molecular Devices Versamax plate reader and analyzed using SOFTmax PRO software. Multiplex cytokine analysis (IL-1α, IL-1β, IL-2, IL-4, IL-6, IL-8, IL-10, VEGF, interferon-γ, EGF, MCP-1, and TNF-α) was performed without replication using the Randox High Sensitivity Cytokine Biochip Array 1 (catalog number EV3623) on the Evidence Investigator analyzer. Detection was by chemiluminescence, with analyte-specific limits of quantification ranging from 0.12 for IL-6 to 1450 ng/L for IL-8, depending on the cytokine. Values below detection limits were reported as the lower limit of detection, and those above upper limits were diluted and reassayed if necessary. In addition, serum NEFA and plasma glucose at KCL were measured using enzymatic colorimetric assays: glucose (Glucose Oxidase method, Werfen Cat No. 00018250840; detection limit: 0.03 mmol/L, upper limit: 41.6 mmol/L) and NEFA (WAKO NEFA-HR Cat No. 434-91795/436-91995; detection limit: 0.01 mmol/L, upper limit: 4 mmol/L) on an ILAB-650 clinical chemistry analyzer (Instrumentation Laboratories). Low, middle, and high-level quality controls were included in all assays. Metabolomics (including apolipoprotein and lipoprotein parameters) were analyzed by high-throughput proton NMR by Nightingale Health, Ltd. A full quality control report was provided by Nightingale Health, confirming high sample integrity and biomarker quantification success rates.

### Flow-mediated vasodilatation and BP

FMD of the brachial artery was measured by ultrasound (Vivid I; GE Healthcare) in a temperature-controlled room. Blood flow was recorded at baseline using the Doppler mode. Assessment of endothelial function was performed using ultrasound echography. For this, a 13-4 MHz linear transducer (MyLab Gamma; Esaote) in B mode was used as described previously [23]. In short, the FMD measurement started with a 3-min reference period. Distal hypoxia was then induced by inflating a pneumatic cuff around the forearm to 200 mm Hg for 5 min, which was followed by a 5-min post occlusive reactive hyperemia response. Brachial artery diameter throughout the FMD measurement was assessed automatically using a custom-written MATLAB program (MyFMD; Prof A.P. Hoeks, Department of Biomedical Engineering, MUMC+, Maastricht, The Netherlands), incorporating automated edge detection, continuous diameter tracking, and use of the true peak diameter for %FMD calculation, in alignment with expert guidelines to minimize measurement error [24]. The FMD response was calculated as the maximal percentage diameter change posthypoxia in relation to the baseline brachial diameter. At MU, FMD was measured in the postprandial subsample (*n* = 12) only.

Office BP was measured 3 times after 10 min rest using an automated clinical digital sphygmomanometer, and the average of the final 2 measurements was calculated.

### Liver fat

The hepatic lipid content was determined by proton magnetic resonance spectroscopy on a 3T magnetic resonance system (Achieva 3T-X; Philips Healthcare) using a STEAM sequence (repetition time of 4500 ms/echo time of 20 ms/mixing time of 16 ms, spectral bandwidth of 2000 Hz, and 2048 data points), and fat content and fat composition was determined as described earlier [25]. Lipid content was calculated after T2 correction as the ratio of the CH2 peak relative to the sum of the unsuppressed water resonance and CH2 peak and converted to weight/weight percentage.

### Statistical analysis

#### Outcomes

The primary outcome variable was change from baseline in total:HDL cholesterol ratio. The choice of total:HDL cholesterol ratio as the primary endpoint reflected its widespread use and recognition as a clinically informative metric at the time of trial design. Secondary outcome variables included fasting and postprandial TAG, non-HDL cholesterol, LDL cholesterol, HDL cholesterol, lipoprotein average diameter, lipoprotein subclass particle concentration, Lp(a), ApoA1, ApoB48, ApoB100, total ApoB, FMD, BP, serum IL-6, IL-8, IL-10, VEGF, TNF-α, IL-1α, IL-1β, MCP-1, and EGF, serum GlycA, plasma glucose, serum insulin, HOMA-IR, serum C-peptide, and NEFA concentrations, body weight, BMI, and waist circumference, liver fat, and dietary energy, macronutrient, and palmitic and stearic acid intakes.

#### Sample size calculations

The sample size calculation for the primary outcome was based on equivalence using an unpaired sample *t* test with an intervention arm difference of 0.09 total:HDL cholesterol ratio, a within-subject SD of differences of 0.2, a 2-sided α of 0.05, and a power of 90%, which would require 48 participants in total. Nonsuperiority was concluded if the dietary intervention arm difference in change exceeded a prespecified equivalence margin of ±0.09. The study aimed to recruit 56 participants, aiming for equal numbers of males and females, allowing for an estimated ∼15% dropout rate, with the target of 48 participants completing the study.

A sample size calculation was also conducted for a secondary outcome, peak postprandial serum TAG concentrations, using an estimated SD of 0.3 mmol/L, expected difference of 0.2 mmol/L, 90% power and α = 0.05, requiring 24 participants to complete the study. A subgroup of 24 (12 at each study center) underwent postprandial test meal challenges at both the beginning and end of each intervention period.

#### Statistical analysis of outcomes

This was performed using IBM SPSS Statistics, version 28.0.1.1. For each of the analyses described below, the assumptions of normality and homogeneity of variance were assessed, and natural log or square root transformation was carried out if necessary. For assessing the chronic effects of dietary intervention, changes from baseline were included as a dependent variable using a linear mixed model. No imputation was performed for missing data. Linear mixed models account for missing data under the assumption that data are missing at random. Terms in the model included intervention arm (repeated effect), visit number (dietary intervention order), intervention arm × visit number, study center, intervention arm × study center, baseline value (covariate), intervention arm × baseline value as fixed effects, and participant identifier as a random effect.

For assessing the chronic effects of dietary intervention on postprandial responses (changes in incremental area under the curve calculated by the trapezoid method, change from fasting or changes in Cmax post minus preintervention) were included as the dependent variable using a linear mixed model. Terms in the model included dietary intervention arm (repeated effect), visit number (dietary intervention arm order), dietary intervention × visit number, baseline value (covariate), dietary intervention × baseline value, study center, and dietary intervention × study center as fixed effects and participant ID as a random effect. To assess changes in the time-course responses, a linear mixed model was run with the timepoint-specific change from baseline value as the dependent variable. Terms in the model included dietary intervention group (repeated effect), visit number (dietary intervention order), dietary intervention × visit number, timepoint (repeated effect), dietary intervention × timepoint, dietary intervention × timepoint × visit, baseline value (covariate), dietary intervention × baseline value, study center, and dietary intervention × study center as fixed effects and participant ID as a random effect. False discovery rate (FDR) adjustment was applied to account for multiple testing where relevant (specified within the tables and results text).

## Results

### Baseline characteristics of the study population

**FIGURE 2:**
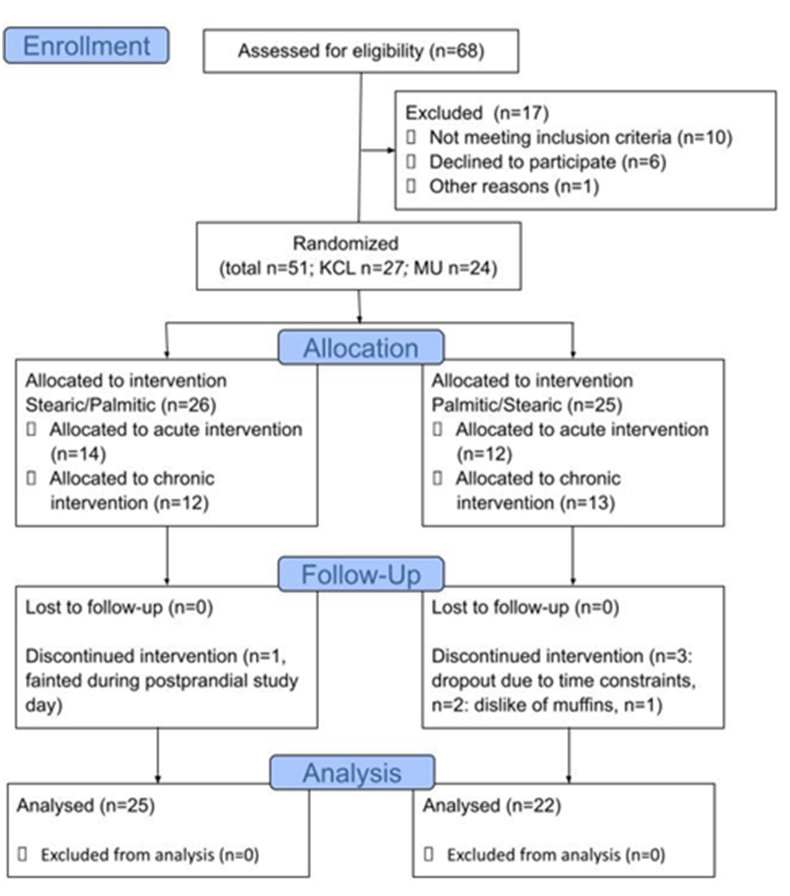
CONSORT flow diagram.

Recruitment initially started in February 2020, but the study at KCL was disrupted by the COVID-19 pandemic, and the study restarted at this center in August 2021. All recruitment was completed in March 2022, and the last participant’s last visit was in August 2022. Across both sites, in total, 68 participants were assessed for eligibility, and 51 were randomly assigned to the intervention arm. A total of 47 subjects completed the study: 23 at KCL and 24 at MU (see CONSORT diagrams, Figure 2 and Supplemental Figures 1 and 2). Baseline characteristics of completed participants are shown in Table 2.

**TABLE 2:** Baseline characteristics of the study population.

	KCL (*n* = 23)	MU (*n* = 24)	Overall (*n* = 47)
Sex (male/female)	11/12	12/12	23/24
Age, y	47.3 (7.9)	55.7 (6.39)	51.6 (8.4)
BMI, kg/m^2^	25.1 (3.39)	25.3 (2.04)	25.2 (2.8)
Body fat, %	26.5 (7.10)	28.3 (5.23)	27.4 (8.5)
Systolic BP, mm Hg	113 (12.4)	123 (12.8)	118 (13.6)
Diastolic BP, mm Hg	76.1 (6.39)	80.5 (7.54)	78.3 (7.4)
Heart rate, bpm	68.3 (10.4)	64.5 (11.3)	66.3 (11.2)
Total cholesterol, mmol/L	5.29 (0.75)	5.71 (0.90)	5.50 (0.85)
HDL cholesterol, mmol/L	1.63 (0.43)	1.78 (0.60)	1.71 (0.51)
LDL cholesterol, mmol/L	3.46 (0.77)	3.46 (0.71)	3.46 (0.74)
TAG, mmol/L	1.16 (0.66)	1.10 (0.45)	1.13 (0.56)
Fasting glucose, mmol/L	5.22 (0.42)	5.06 (0.43)	5.14 (0.43)

Values are presented as mean (SD).

Abbreviations: BMI, body mass index; BP, blood pressure; bpm, beats per minute; HDL, high-density lipoprotein; KCL, King’s College London; MU, Maastricht University; LDL, low-density lipoprotein; TAG, triacylglycerol.

### Compliance with intervention

**FIGURE 3:**
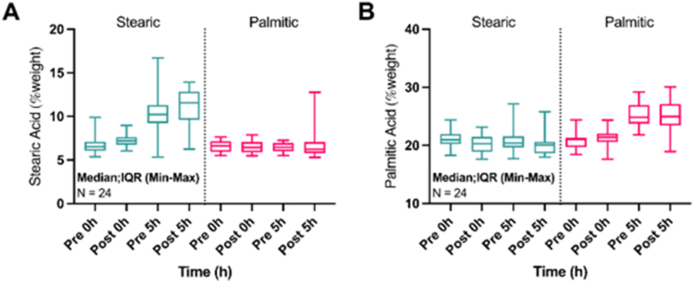
Plasma proportions of (A) stearic acid (18:0) and (B) palmitic acid (16:0) at fasting (0 h) and postprandially at 4.9 h at baseline and endpoint following 6 weeks of daily test fat consumption. Statistical analysis used linear mixed models: changes from baseline as a dependent variable; fixed effects included dietary intervention group (repeated effect), visit number (dietary intervention order), dietary intervention × visit number, center, dietary intervention × center, baseline value (covariate), dietary intervention × baseline value; and participant ID was included as a random effect. Significant differences between dietary interventions for changes in stearic acid and palmitic acid proportions in total plasma were noted at fasting and postprandially. Box plots represent medians with interquartile ranges, and whiskers represent the minimum and maximum values.

Dietary intakes and the contribution of the test fats to fatty acid intakes at the end of each intervention are shown in Table 3. Both stearic acid and palmitic acid intakes were significantly different between Stearic and Palmitic interventions at the endpoint (*P* < 0.001). Mean differences in palmitic acid intake (% energy) after Stearic compared with Palmitic interventions were −3.9 ± 0.2% at MU (assessed by FFQ) and −3.2 ± 0.9 % at KCL (assessed by 4-d food diary). Mean differences in stearic acid intake (% energy) after Stearic compared with Palmitic interventions were 3.2% ± 0.2% and 2.3% ± 0.7% at MU and KCL, respectively. Experimental fats contributed 4.6% ± 0.3% and 3.6% ± 1.0% energy as palmitic acid in the Palmitic intervention arm in MU and KCL, respectively, and 3.7% ± 0.2% and 2.9% ± 0.8% energy as stearic acid in the Stearic intervention arm. Fasting plasma concentrations (% weight) of stearic acid increased significantly after the Stearic compared with Palmitic interventions (mean difference in change from baseline: 0.81% weight; 95% confidence interval [CI]: 0.62, 1.00%; *P* < 0.001). Likewise, fasting concentrations of palmitic acid increased significantly after the Palmitic compared with Stearic interventions (mean difference in change from baseline: 1.23% weight; 95% CI: 0.81, 1.65%; *P* < 0.001). Postprandial concentrations of palmitic and stearic acids increased significantly at 5 h after the palmitic and stearic acid-rich test meals, respectively, *P* < 0.001 (Figure 3).

**TABLE 3:** Dietary energy, macronutrient, palmitic and stearic acid intakes at endpoint following 6-wk dietary intervention with interesterified test fats rich in stearic or palmitic acids.

	KCL (*n* = 23)				MU (*n* = 24)			
	Stearic	Palmitic	Δ Stearic − Palmitic	*P*	Stearic	Palmitic	Δ Stearic − Palmitic	*P*
Energy, MJ	9.3 (2.9)	8.8 (1.9)	0.5 (2.4)	0.360	9.2 (1.3)	9.1 (1.2)	0.1 (0.4)	0.249
Total fat, %E	35.2 (6.5)	36.6 (6.1)	−1.3 (7.8)	0.427	41.2 (4.9)	40.4 (4.8)	0.8 (1.9)	0.046
Total fat from test fats	7.6 (2.2)	7.7 (2.3)	−0.1 (2.1)	0.784	11.2 (0.8)	11.3 (0.8)	−0.1 (0.4)	0.332
Total SFA, %E	14.0 (3.6)	13.6 (2.6)	0.4 (3.4)	0.564	17.3 (2.2)	16.6 (2.1)	0.7 (0.5)	<0.001
Total SFA from test fats	5.9 (1.8)	5.4 (1.6)	0.6 (1.6)	0.114	7.2 (0.4)	6.6 (0.4)	0.5 (0.3)	<0.001
Total palmitic acid (16:0) from test fats	0.4 (0.1)	3.6 (1.0)	−3.2 (0.9)	<0.001	0.7 (0.0)	4.6 (0.3)	−3.9 (0.2)	<0.001
Total stearic acid (18:0) from test fats	2.9 (0.8)	0.6 (0.2)	2.3 (0.7)	<0.001	3.7 (0.2)	0.5 (0.0)	3.2 (0.2)	<0.001
Total carbohydrates, %E	46.6 (6.5)	44.9 (7.1)	1.8 (8.3)	0.334	41.3 (5.1)	41.9 (5.3)	−0.6 (1.6)	0.084
Total sugars, %E	15.1 (4.7)	15.6 (4.6)	−0.5 (3.0)	0.431	16.7 (3.7)	17.0 (3.8)	−0.4 (1.0)	0.109
Total protein, %E	16.2 (2.3)	16.7 (4.1)	−0.5 (3.7)	0.499	14.0 (1.9)	14.2 (1.9)	−0.2 (0.3)	0.016

Values are presented as mean (SD). At KCL, dietary intakes were assessed by 4-d food diary; data for 1 subject is missing. At MU, dietary intakes were assessed by FFQ; data for 1 subject is missing. The difference between dietary interventions (Δ Stearic − Palmitic) was compared using a paired sample *t* test.

Abbreviations: KCL, King’s College London; MJ, megajoules; MU, Maastricht University; SFA, saturated fatty acid.

### Total-to-HDL cholesterol ratio

There were no significant differences in fasting total:HDL cholesterol ratio between interventions at the endpoint (Table 4). There were no differences between baseline and endpoint measures within arms (after FDR adjustment), nor were there any differences at baseline between interventions.

**TABLE 4:** Changes in body weight, waist circumference, fasting lipids, endothelial function, liver fat, inflammatory markers, and fasting serum cytokines at baseline and after 6-wk dietary intervention (stearic or palmitic).

Outcome	Baseline Stearic Mean (95% CI)	Δ Stearic Mean (95% CI) Median (Q1, Q3)^1^	Baseline Palmitic Mean (95% CI)	Δ Palmitic Mean (95% CI) Median (Q1, Q3)^1^	Difference (Δ Stearic – Δ Palmitic) Mean (95% CI) Median (Q1, Q3)^1^
Body weight, kg	76.0 (72.0, 80.0)	0.21 (−0.10, 0.52)	75.6 (71.5, 79.6)	0.10 (−0.35, 0.54)	0.11 (−0.46, 0.68)
Waist, cm	90.8 (87.7, 93.9)	0.01 (−0.85, 0.87)	90.5 (87.3, 93.6)	−0.46 (−1.09, 0.17)	0.47 (−0.48, 1.42)
SBP, mm Hg	119.6 (115.4, 123.8)	−1.51 (−3.92, 0.89)	117.2 (113.7, 120.7)	−0.76 (−3.33, 1.80)	−0.75 (−4.45, 2.95)
DBP, mm Hg	77.8 (75.5, 80.2)	−0.91 (−1.80, 1.61)	77.1 (75.1, 79.0)	−0.06 (−1.85, 1.73)	−0.03 (−2.06, 2.01)
Total-C: HDL-C	3.55 (3.30, 3.81)	0.03 (−0.063, 0.118)	3.51 (3.25, 3.77)	−0.03 (−0.107, 0.056)	0.053 (−0.077, 0.184)
Total-C, mmol/L	5.37 (5.10, 5.70)	−0.032 (−0.170, 0.150)	5.37 (5.05, 5.64)	0.00 (−0.171, 0.171)	−0.032 (−0.229, 0.164)
LDL-C, mmol/L	3.57 (3.31, 3.84)	0.012 (−0.125, 0.150)	3.57 (3.29, 3.85)	0.001 (−0.151, 0.153)	0.011 (−0.197, 0.220)
TAG, mmol/L	0.97 (0.86, 1.10)^2^	−0.001 (−0.093, 0.091)	0.94 (0.84, 1.05)^2^	−0.002 (−0.075, 0.071)	−0.001 (−0.106, 0.108)
HDL-C, mmol/L	1.63 (1.48, 1.78)	−0.022 (−0.066, 0.021)	1.64 (1.50, 1.79)	0.012 (−0.030, 0.053)	−0.034 (−0.091, 0.023)
Non-HDL-C, mmol/L	3.83 (3.58, 4.09)	−0.011 (−0.134, 0.113)	3.81 (3.54, 4.09)	−0.015 (−0.160, 0.131)	0.004 (−0.180, 0.188)
Ln(apoB48)^3^, mg/L	2.73 (2.52, 2.95)	−0.119 (−0.255, 0.018)	2.68 (2.47, 2.89)	0.034 (−0.061, 0.129)	−0.152 (−0.313, 0.008)
Ln(apoB100)^3^, mg/L	7.04 (6.92, 7.15)	0.009 (−0.044, 0.062)	7.03 (6.92, 7.14)	−0.043 (−0.095, 0.009)	0.052 (−0.022, 0.126)
Ln(Lp(a))^3^, mg/L	5.73 (5.50, 5.96)	−0.026 (−0.125, 0.073)	5.69 (5.46, 5.92)	−0.084 (−0.167, −0.001)	0.058 (−0.058, 0.174)
NEFA, mmol/L	0.42 (0.36, 0.49)	−0.028 (−0.08, 0.02)	0.47 (0.39, 0.55)	−0.011 (−0.06, 0.04)	−0.017 (−0.091, 0.057)
Glucose, mmol/L	5.68 (5.49, 5.87)	0.065 (−0.041, 0.172)	5.56 (5.33, 5.78)	−0.080 (−0.224, 0.064)	0.146 (−0.025, 0.316)
Insulin, mIU/L	7.63 (6.44, 8.82)	0.175 (−0.448, 0.797)	7.29 (6.30, 8.29)	0.027 (−0.818, 0.873)	0.147 (−0.722, 1.016)
HOMA-IR	2.02 (1.71, 2.33)	0.044 (−0.129, 0.216)	1.88 (1.53, 2.22)	−0.013 (−0.265, 0.240)	0.056 (−0.245, 0.358)
C-peptide, μg/L	1.39 (1.21, 1.57)	−0.008 (−0.075, 0.059)	1.34 (1.20, 1.48)	−0.009 (−0.096, 0.077)	0.001 (−0.096, 0.099)
FMD^4^, %	5.33 (4.04, 6.61)	−0.94 (−1.039, 0.852)	6.14 (4.94, 7.32)	−0.527 (−1.410, 0.355)	0.434 (−0.881, 1.749)
Liver fat, %	1.420 (0.847, 2.378)^2^	0.212 (−0.076, 0.500)	1.415 (0.810, 2.471)^2^	−0.090 (−0.608, 0.428)	0.302 (−0.208, 0.811)
GlycA, mmol/L	0.76 (0.73, 0.79)	−0.008 (−0.027, 0.010)	0.76 (0.73, 0.79)	0.002 (−0.016, 0.019)	−0.010 (−0.035, 0.015)
IL-6, ng/L	1.68 (1.17, 2.32)	0.03 (−0.06, 0.40)^1^	1.61(1.06, 2.31)	−0.05 (−0.42, 0.19)^1^	−0.01 (−0.13, 0.14)^1^^,^^5^
IL-8, ng/L	12.12 (10.13, 14.47)	0.17 (−1.36, 1.13)^1^	11.28 (9.12, 13.90)	−0.26 (−1.59, 1.09)^1^	−0.02 (−0.13, 0.10)^1^
IL-10, ng/L	1.10 (0.59, 1.79)	0.00 (−0.11, 0.23)^1^	0.89 (0.74, 1.05)	−0.06 (−0.17, 0.13)^1^	0.14 (0.06, 0.23)^1^^,^^6^
VEGF, ng/L	56.65 (42.38, 75.63)	−1.01 (−4.38, 9.33)^1^	58.07 (43.02, 78.27)	−2.02 (−14.03, 2.61)^1^	0.08 (−0.05, 0.22)^1^
TNF-α, ng/L	3.25 (2.74, 3.83)	−0.07 (−0.21, 0.30)^1^	3.15 (2.68, 3.68)	−0.27 (−0.88, 0.2)^1^	0.06 (−0.03, 0.17)^1^
IL-1α, ng/L	0.38 (0.28, 0.49)	−0.03 (−0.08, 0.05)^1^	0.40 (0.27, 0.54)	0.00 (−0.05, 0.07)^1^	−0.02 (−0.07, 0.03)^1^
IL-1β, ng/L	4.05 (2.88, 5.58)	−0.35 (−1.36, 0.75)^1^	3.84 (2.61, 5.49)	0.07 (−1.07, 0.66)^1^	−0.09 (−0.24, 0.10)^1^
MCP-1, ng/L	228.4 (189.8, 274.8)	−2.86 (−24.70, 29.12)^1^	221.3 (181.0, 270.4)	−0.24 (−26.65, 19.65)^1^	0.05 (−0.04, 0.15)^1^
EGF, ng/L	21.6 (14.41, 32.07)	4.12 (−6.01, 9.36)^1^	20.5 (13.98, 29.92)	1.50 (−5.05, 23.96)^1^	−0.10 (−0.43, 0.41)^1^

apoB48: Stearic, baseline 16.67 mg/L (20.45), change −0.64 mg/L (6.03); Palmitic, baseline 15.07 mg/L (14.71), change mg/L 0.46 (4.42).

apoB100: Stearic, baseline 1080.5 mg/L (709.0), change −7.5 mg/L (230.0); Palmitic, baseline 1152.0 mg/L (681), change −17.0 mg/L (232.0).

Lp(a): Stearic, baseline 291.0 mg/L (323.0), change −1.5 mg/L (64.0); Palmitic, baseline 272.5 mg/L (1012.0), change −23.5 (69.0).

Liver fat: Stearic, baseline 1.33% (1.19), change 0.24% (0.64); Palmitic, baseline 1.28% (1.54), change −0.15% (0.89).

Abbreviations: Apo, apolipoprotein; DBP, diastolic blood pressure; EGF, epidermal growth factor; GlycA, glycoprotein acetyls; FMD, flow-mediated dilatation; HDL-C, high-density lipoprotein cholesterol; HOMA-IR, homeostatic model assessment for insulin resistance; IL, interleukin; LDL-C, low-density lipoprotein cholesterol; Lp(a), lipoprotein (a); MCP-1, monocyte chemoattractant protein 1; NEFA, non-esterified fatty acid; SBP, systolic blood pressure; TAG, triacylglycerol; TNF-α, tumor necrosis factor α; Total-C, total cholesterol; VEGF, vascular endothelial growth factor.

Values are presented as mean (95% CI) or median (Q1, Q3) where indicated. Statistical analysis was conducted by linear mixed models including changes from baseline as the dependent variable, intervention arm (repeated effect), visit number (dietary intervention order), intervention arm × visit number, study center, intervention arm × study center, baseline value (covariate), intervention arm × baseline value as fixed effects, and participant identifier as a random effect. There were no significant differences between dietary interventions. An equivalence margin of ±0.09 was prespecified for the total-C: HDL-C ratio. Nonsuperiority was concluded if the intervention arm difference in change exceeded this range.

1 Medians (with lower and upper limits of IQR) are provided where denoted.

2 Back transformed.

3 Natural log transformed values shown.

4 *n* = 35, conducted on all KCL participants (*n* = 23) and a subset of MU participants (*n* = 12).

5 Residuals of mixed model nonnormal so data presented as median (IQR) and difference in median change between dietary interventions assessed by Wilcoxon rank sum test.

6 False discovery rate adjusted *P* < 0.01.

### Peak postprandial TAG concentrations

For postprandial peak TAG concentrations (Cmax), there were no intervention effects on change in peak postprandial TAG (Cmax), calculated as endpoint minus baseline (mean difference: −0.22 mmol/L; 95% CI: −0.59, 0.15).

### Fasting cardiometabolic risk measures

There were no significant differences in any fasting outcome variables between interventions at baseline or endpoint (Table 4 and Supplemental Table 2), nor were there any differences between baseline and endpoint measures within arms (after FDR adjustment). However, relative to the change after the Stearic intervention, there was a decrease in the anti-inflammatory cytokine, IL-10, after the Palmitic intervention; geometric mean: −0.14 (95% CI: −0.23, −0.06).

### Postprandial cardiometabolic risk measures

**FIGURE 4:**
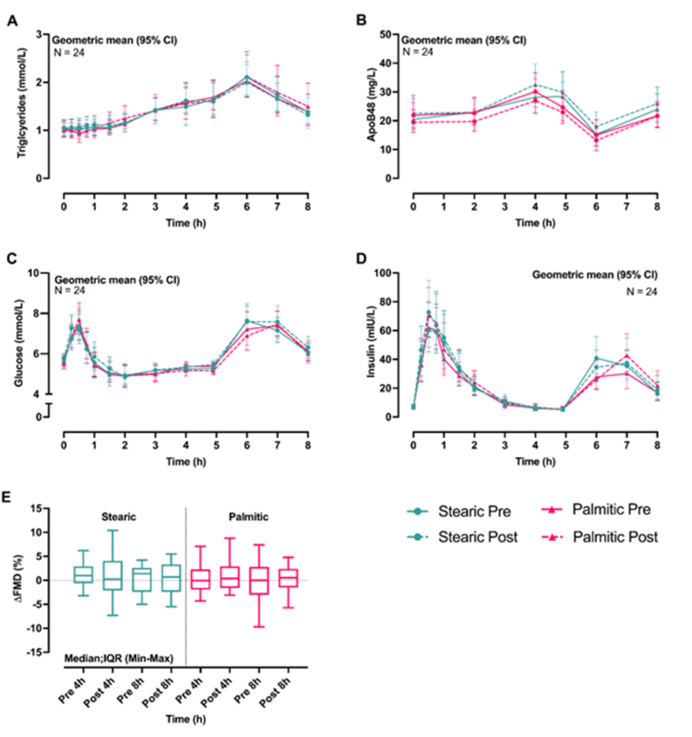
Postprandial change in (A) plasma TAG over 8 h; (B) apoB48 over 8 h; (C) plasma glucose over 8 h; (D) serum insulin over 8 h; (E) FMD at 4 h and 8 h following test meals containing fat rich in stearic acid or palmitic acid shown both at baseline (pre) and following (post) 6-wk dietary intervention (stearic or palmitic; *n* = 24). Statistical analysis used linear mixed models. (A-D) Changes from baseline as a dependent variable; fixed effects included dietary intervention group (repeated effect), visit number (dietary intervention order), dietary intervention × visit number, timepoint (repeated effect), dietary intervention × timepoint, dietary intervention × timepoint × visit, baseline value (covariate), dietary intervention × baseline value, center, dietary intervention × center; and participant ID was included as a random effect. No significant differences were observed either between dietary interventions or within arms. (E) Changes from baseline as a dependent variable; fixed effects included dietary intervention group (repeated effect), visit number (dietary intervention order), center, dietary intervention × center, dietary intervention × visit number, baseline value (covariate), dietary intervention × baseline value; and participant ID was included as a random effect. Box plots represent medians with interquartile ranges, and whiskers represent the minimum and maximum values.

Postprandial measures of TAG, ApoB48, glucose, insulin, and FMD (Figure 4A–E), lipoprotein subclass particle concentrations (Table 5), and ApoB, ApoA1, average diameters of HDL, LDL, and VLDL, NEFA, C-peptide, and inflammatory markers (Supplemental Figures 3–7 and Supplemental Table 3) were measured ≤8 h at baseline and endpoint of each intervention arm in a subset of 24 participants (MU, *n* = 12; KCL, *n* = 12). There were no significant differences in postprandial responses in any of the postprandial measures reported below, between interventions or within intervention arms (baseline compared with endpoint).

**TABLE 5:** Postprandial serum lipoprotein particle concentration incremental area under the curve (iAUC 0–8 h) after high-fat test meals rich in stearic or palmitic acid, shown both at baseline, endpoint, and changes after 6-wk dietary intervention.

iAUC	Stearic (nmol·h/L)			Palmitic (nmol·h/L)			Mean Difference^1^ (Δ Stearic – Δ Palmitic) Geomean (95% CI) (pmol·h/L)
	Baseline	Endpoint	Δ	Baseline	Endpoint	Δ	
	Geomean (95% CI)	Geomean (95% CI)	Median (IQR)	Geomean (95% CI)	Geomean (95% CI)	Median (IQR)	
XL-HDL-P	0.2 (0.1, 0.2)	0.2 (0.1, 0.2)	0.0 (−0.0, 0.1)	0.2 (0.1, 0.2)	0.2 (0.1, 0.2)	0.00 (−0.1, 0.1)	0.01 (−0.03, 0.005)
L-HDL-P	0.9 (0.6, 1.2)	0.9 (0.7, 1.1)	0.0 (−0.4, 0.3)	1.0 (0.7, 1.2)	1.0 (0.8, 1.3)	0.05 (−0.1, 0.2)	−0.1 (−0.4, 0.2)
M-HDL-P	1.8 (1.2, 2.3)	1.3 (0.9, 1.7)	−0.7 (−1.3, 0.2)	1.6 (1.1, 2.0)	2.0 (1.5, 2.5)	0.2 (−0.1, 1.1)	−1.0 (−2.0, −0.2)
S-HDL-P	1.4 (0.6, 2.3)	0.4 (0.1, 0.7)	−0.4 (−2.2, 0.0)	0.6 (0.3, 0.9)	1.1 (0.5, 1.7)	0 (−0.2, 0.9)	−1.4 (−3.0, −0.3)
L-LDL-P	0.06 (0.02, 0.10)	0.06 (0.01, 0.10)	0 (−0.03, 0.01)	0.0 (0.01, 0.10)	0.08 (0.03, 0.12)	0.01 (−0.0001, 0.07)	−0.02 (−0.1, 0.1)^2^
M-LDL-P	0.08 (0.05, 0.10)	0.05 (0.02, 0.09)	−0.01 (−0.1, 0.03)	0.07 (0.03, 0.10)	0.09 (0.06, 0.10)	0.005 (−0.04, 0.10)	−0.04 (−0.1, 0.04)^2^
S-LDL-P	0.03 (0.02, 0.05)	0.02 (0.01, 0.03)	−0.01 (−0.04, 0.002)	0.02 (0.01, 0.04)	0.04 (0.01, 0.05)	0.01 (−0.01, 0.04)	−0.04 (−0.1, 0.01)^2^
XXL-VLDL/chylo-P	0.01 (0.00, 0.01)	0.007 (0.005, 0.009)	−0.001 (−0.004, 0.001)	0.008 (0.006, 0.01)	0.009 (0.007, 0.013)	0.001 (−0.001, 0.003)	−0.003 (−0.01, −0.0004)
XL-VLDL-P	0.01 (0.008, 0.01)	0.009 (0.007, 0.012)	−0.001 (−0.004, 0.001)	0.01 (0.007, 0.013)	0.01 (0.009, 0.016)	0.001 (−0.002, 0.005)	−0.003 (−0.01, 0.0001)
L-VLDL-P	0.02 (0.016, 0.03)	0.022 (0.015, 0.029)	−0.00002 (−0.007, 0.004)	0.022 (0.016, 0.028)	0.027 (0.018, 0.036)	0.003 (−0.004, 0.013)	−0.006 (−0.01, 0.003)
M-VLDL-P	0.022 (0.013, 0.031)	0.022 (0.013, 0.031)	−0.001 (−0.01, 0.01)	0.017 (0.009, 0.025)	0.024 (0.013, 0.034)	0.002 (−0.003, 0.016)	−0.006 (−0.002, 0.01)
S-VLDL-P	0.03 (0.02, 0.04)	0.03 (0.02, 0.04)	−0.005 (−0.016, 0.017)	0.022 (0.013, 0.031)	0.029 (0.018, 0.041)	0.002 (−0.004, 0.016)	−0.005 (−0.002, 0.01)
XS-VLDL-P	0.02 (0.01, 0.03)	0.03 (0.02, 0.04)	0.001 (−0.004, 0.011)	0.023 (0.012, 0.034)	0.024 (0.016, 0.032)	0.0004 (−0.004, 0.01)	0.004 (−0.01, 0.002)

Abbreviations: chylo-P, chylomicron particle; L-HDL-P, large high-density lipoprotein particle; L-LDL-P, large low-density lipoprotein particle; L-VLDL-P, large very low-density lipoprotein particle; M-HDL-P, medium high-density lipoprotein particle; M-LDL-P, medium low-density lipoprotein particle; M-VLDL-P, medium very low-density lipoprotein particle; S-HDL-P, small high-density lipoprotein particle; S-LDL-P, small low-density lipoprotein particle; S-VLDL-P, small very low-density lipoprotein particle; XL-HDL-P, extra-large high-density lipoprotein particle; XL-VLDL-P, extra-large very low-density lipoprotein particle; XS-VLDL-P, extra-small very low-density lipoprotein particle; XXL-VLDL-P, extra-extra-large very low-density lipoprotein particle, Δ = endpoint – baseline.

*n* = 24 included in analysis. Statistical analysis was conducted by linear mixed models including changes in iAUC (0–8 h) calculated by the trapezoid method as the dependent variable; dietary intervention arm (repeated effect), visit number (dietary intervention arm order), dietary intervention × visit number, baseline value (covariate), dietary intervention × baseline value, study center, dietary intervention × study center as fixed effects; and participant ID as a random effect.

1 Estimated marginal means from linear mixed model.

2 Residuals of mixed model nonnormal so data presented as median (IQR) and difference in median change between dietary interventions assessed by Wilcoxon rank sum test.

## Discussion

This study aimed to compare the cardiometabolic health effects of commonly consumed stearic and palmitic acid-rich IE fats in a randomized crossover trial. It was hypothesized that a commercially relevant palmitic acid-rich IE fat would have effects equivalent to those of a functionally matched stearic acid-rich IE fat on total:HDL cholesterol ratio, within a predefined equivalence margin of ±0.09, as well as on other chronic and acute intermediary CMD risk markers, when consumed at achievable upper levels of dietary intake.

In agreement with this hypothesis, no significant acute or chronic differences were found in circulating lipid, apolipoproteins, lipoproteins, endothelial function, inflammatory markers, glucose homeostasis, or insulin resistance between intervention arms. These findings are relevant as they indicate that realistic intake levels of two commonly used hardstock fats, one formulated with palmitic acid-rich palm fat and the other with a stearic acid-rich blend of fully hydrogenated seed and coconut oil, had no detectable effect on lipid metabolism during an intervention of sufficient duration to observe expected changes.

The study aimed to provide 10% of estimated energy requirements from the test fats, contributing an additional 5% and 4% energy from palmitic acid and stearic acid in the Palmitic and Stearic arms, respectively. These targets were largely achieved, as reflected in average energy intake from the test fats and supported by significant increases in circulating palmitic acid and stearic acid postintervention. Although minor differences in self-reported test fat intakes were observed between study centers, statistical analysis found no center effect on intervention outcomes. These discrepancies likely reflect differences in dietary assessment methods, a limitation of the study.

Equivalence in effects on total:HDL cholesterol ratio is plausible given the greater LDL cholesterol- and HDL cholesterol-raising effect of palmitic acid compared with stearic acid, such that overall changes in the ratio may be similar [26]. Minor differences in other fatty acids, including the LDL cholesterol-raising myristic and lauric acids, were present but small (lauric acid: 1.6% compared with 0.9% energy; myristic acid: 0.6% compared with 0.4% energy in the Stearic and Palmitic arms, respectively).

Previous studies [27,28] showing lipid differences between high palmitic acid and high stearic acid diets typically involved larger intake differentials (>6% energy) than the present trial. For example, a prior 4-wk intervention study by some of the present authors [29] comparing diets with a difference in intakes of stearic acid and palmitic acid of 6.0% and 6.5%, respectively, reported significantly lower total cholesterol, LDL cholesterol, HDL cholesterol, ApoA1, and postprandial TAG concentrations after the stearic acid-rich diet. Interestingly, inflammatory cytokines IL-6 and TNF-α (but not high sensitivity C-reactive protein) and total:HDL cholesterol ratio were higher after the stearic acid-rich diet, despite lipid reductions [29]. These contrasting findings may reflect differences in test fat blends, absence of IE, and greater intake differentials. A systematic review concluded that although IE of palmitic and stearic acid-rich fats did not affect serum lipids overall, substitution of palmitic acid with stearic acid lowered LDL cholesterol [30].

High-fat meals can acutely impair endothelial function, likely via reduced nitric oxide bioavailability due to oxidative stress and proinflammatory cytokines [31]. However, in this study, realistic intakes of stearic or palmitic acid-rich IE fats had no differential effect on fasting inflammatory cytokines or endothelium-dependent vasodilation. Although dietary SFAs are linked with higher inflammatory markers [32], and animal/in vitro studies show proinflammatory effects of palmitic acid-rich fats [33], human studies comparing palmitic acid and stearic acid on inflammation are inconsistent [28,34]. Notably, concentrations of IL-10 (an anti-inflammatory cytokine) were reduced by the Palmitic intervention relative to the Stearic. This finding warrants further investigation to assess whether stearic acid-rich fats are relatively neutral in inflammatory effects and whether this has clinical relevance.

This study also explored postprandial responses, given that nonfasting TAG concentrations are associated with increased CVD risk [[35], [36], [37]], potentially mediated by inflammatory responses to high-fat meals [38,39] and endothelial dysfunction [[40], [41], [42]]. As expected, postprandial increases in TAG, lipoprotein subclasses, apolipoproteins, and some inflammatory markers were observed 0 to 8 h after test meals, but no intervention or time (pre/post) effects were seen. Previous studies (summarized elsewhere [30]) have reported inconsistent findings on postprandial differences between stearic and palmitic acid-rich fats and between IE and non-IE fats. These discrepancies may relate to differences in solid fat content at 37°C [43]. Our group’s recent work showed no postprandial TAG difference between IE and non-IE palm-based fats (80:20 palm stearin/palm kernel), despite differences in positional fatty acid composition [44]. This likely reflects similar solid fat content at body temperature [44]. Indeed, the study by our group discussed earlier [29], which reported a significantly lower postprandial TAG and ApoB48 response after consumption of stearic acid-rich compared with palmitic acid-rich fat [45], used test fats with a difference in solid fat content at 37°C (1% and 8%, respectively). The current study intentionally used fats matched for functional performance and solid fat content at 37°C, which may explain the lack of difference in postprandial responses.

Using the United Kingdom National Diet and Nutrition Survey (years 5–6, 2012–2014), we estimated average IE intake at 1.1% energy (95% CI: 0, 3.45%) [46], lower than United States NHANES estimates (∼3% energy, upper limit of 4.8%) based on assumed replacement of *trans* fats [47]. However, not all *trans* fats have been replaced by IE fats, making 1% to 2% energy a plausible estimate for current intakes. NHANES modeling predicted that IE fat substitution would increase palmitic and stearic acid intakes by 1% to 2% and 0.5% to 1.5% energy, respectively. The present study exceeded these average increases (∼3%–5% energy from palmitic or stearic acid), but remained below estimated mean intakes for the highest quintile (4.8% and 3.8% energy for palmitic and stearic acid, respectively) [47]. Thus, our results are applicable to a wide population range.

A key strength of this study is the use of commercially relevant, functionally equivalent fats delivered at realistic dietary doses, exceeding current population-level exposures in the United Kingdom [46]. However, some limitations must be noted. Although the study was adequately powered for equivalence testing using a margin of ±0.09 on the primary outcome (total:HDL cholesterol ratio) and for differences in the secondary outcome (peak postprandial TAG), it may have been underpowered for other endpoints. Furthermore, whereas total:HDL cholesterol ratio is historically regarded as one of the strongest predictors of CVD risk [14,26] and more recently has been shown to remain discriminatory at lower LDL cholesterol and non-HDL cholesterol concentrations [15], causal evidence supports LDL cholesterol and triglyceride-rich lipoproteins (components of non-HDL cholesterol) as direct mediators of atherosclerotic risk [48]. Although HDL cholesterol is inversely associated with CVD risk, interventions and genetic studies have not demonstrated a causal effect of raising HDL cholesterol. Therefore, results based on the total:HDL cholesterol ratio should be interpreted alongside findings for LDL cholesterol and non-HDL cholesterol, which were consistent in supporting equivalence of the two interventions. The sample size was insufficient to stratify analyses by sex, menopause status, age, or BMI—factors that influence individual cardiometabolic responses to dietary fat. Males and females may respond differently to dietary interventions; therefore, we cannot exclude the possibility of sex-specific effects, which should be further explored in future studies. Although factors such as genetic variation, ω-3 status, gut microbiota, and physical activity may modulate individual responses, the randomized crossover design helped control interindividual variability. The study was conducted in healthy participants in the United Kingdom and The Netherlands within a specific age range, and therefore, the results cannot be extrapolated to those with CMDs or other population groups. A key limitation is the relatively short 6-week intervention period. Although this duration has previously been sufficient to detect changes in lipid and vascular markers [[49], [50], [51]], longer-term effects remain unknown. Finally, dietary fatty acid intakes from other meals were not controlled. However, a tightly controlled metabolic feeding study would not have been feasible for a total duration of 18 weeks.

In conclusion, at realistic intake levels, consuming commercially relevant IE fat blends rich in either palmitic or stearic acid does not differentially affect total:HDL cholesterol ratio, other lipids, endothelial function, inflammation, or insulin resistance, either acutely or after 6 weeks. These findings suggest that formulating processed foods with either palmitic or stearic acid-rich IE fats is unlikely to have adverse cardiometabolic consequences at intakes ≤10% energy. This study provides essential evidence to inform oils and fats manufacturers, the food industry, health authorities, and health care professionals on the cardiometabolic safety of IE fats used in commonly consumed products.

## Author contributions

The authors’ responsibilities were as follows – WLH, HAS: conducted statistical analysis; SEB, WLH: wrote the initial manuscript draft and finalized the submitted version; SEB, RPM: conceptualized the study and acquired funding; SEB, RPM, WLH, PJJ: supervised research activity planning and execution, designed the trials, interpreted data, and reviewed and edited subsequent versions of the manuscript; EW, PJJ, AC, TM: conducted the clinical trial and collected the data; JHB: formulated the test fats and spreads, provided test fat compositional data, and provided advice on the design of the dietary intervention; and all authors: commented on the initial manuscript draft and read and approved the final manuscript.

## Data availability

Data described in the manuscript, code book, and analytic code will be made available upon request pending application and approval.

## Funding

This research was funded by a research grant from the Malaysian Palm Oil Board. The funders were not involved in the study design; collection, analysis, and interpretation of data; writing of the report; or restrictions regarding publication.

## Conflict of interest

Sarah Berry reports financial support was provided by Malaysian Palm Oil Board. Harry Smith reports a relationship with Zoe Ltd that includes: equity or stocks. Sarah Berry reports a relationship with Zoe Ltd that includes: consulting or advisory, equity or stocks, and travel reimbursement. Wendy Hall reports a relationship with Zoe Ltd that includes: consulting or advisory. Alice Creedon reports a relationship with Zoe Ltd that includes: employment. Wendy Hall reports a relationship with Almond Board of California that includes: funding grants. Johanna Bruce reports a relationship with ADM Ltd that includes: employment. Peter Joris reports a relationship with Peanut Institute Foundation that includes: funding grants. Ronald Mensink reports a relationship with Peanut Institute Foundation that includes: funding grants. Peter Joris reports a relationship with Wild Blueberry Association of North America that includes: funding grants. Peter Joris reports a relationship with Cosun Nutrition Center that includes: funding grants. Peter Joris reports a relationship with California Walnut Commission that includes: funding grants. Co-author was previously employed by Zoe Ltd (HAS). If there are other authors, they declare that they have no known competing financial interests or personal relationships that could have appeared to influence the work reported in this paper.
