# Supplementary material for: The effects of consumption of interesterified fats rich in palmitic acid compared with stearic acid on intermediary markers of cardiometabolic disease risk: a randomized controlled trial in healthy adults
Source: Am J Clin Nutr. 2025 Sep 18;122(5):1361–73. doi: 10.1016/j.ajcnut.2025.09.025 (PMC12799373; doi:10.1016/j.ajcnut.2025.09.025)
Supplement: Multimedia component 1 [file mmc1.docx]

**The effects of consumption of interesterified fats rich in palmitic acid versus stearic acid on intermediary markers of cardiometabolic disease risk: a randomized controlled trial in healthy adults.**

Wendy L. Hall, Eleanor Wood, Peter J. Joris, Harry A. Smith, Alice Creedon, Tyler Maher, Johanna H Bruce, Ronald P. Mensink & Sarah E. Berry

**Supplementary Material**


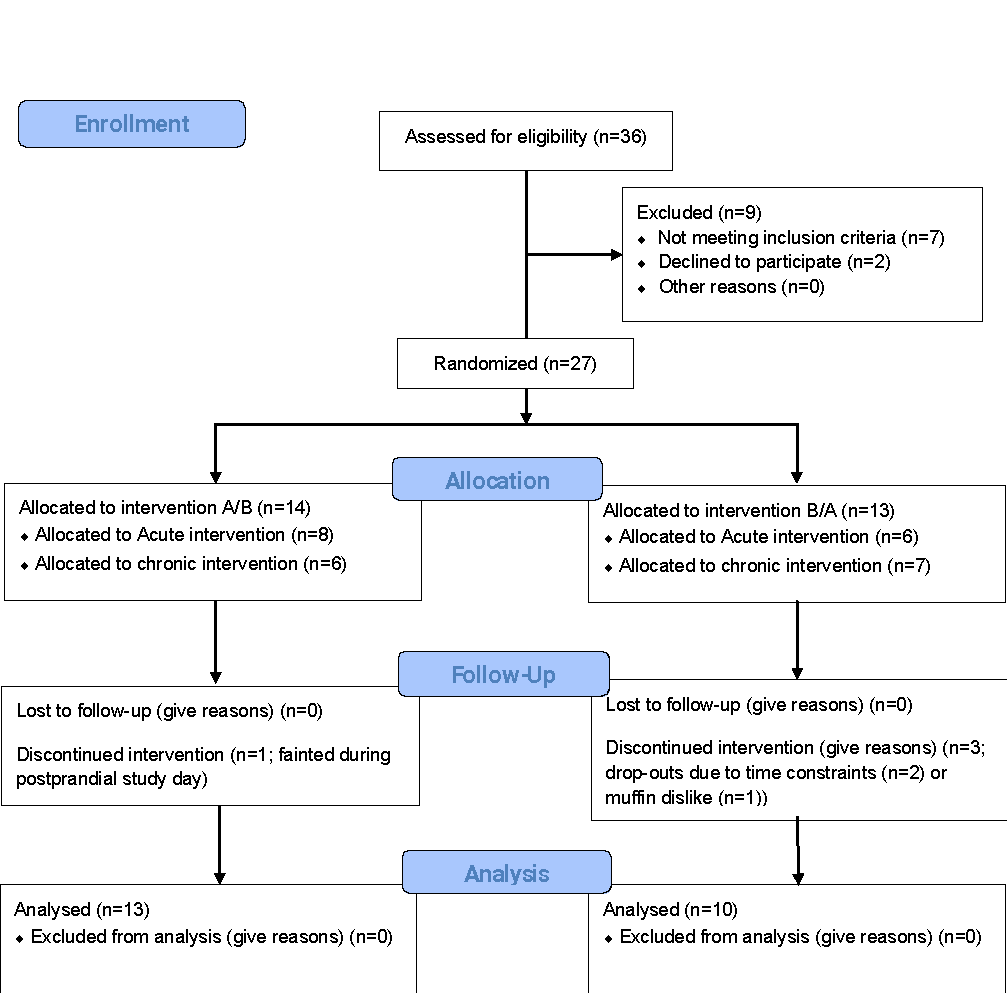

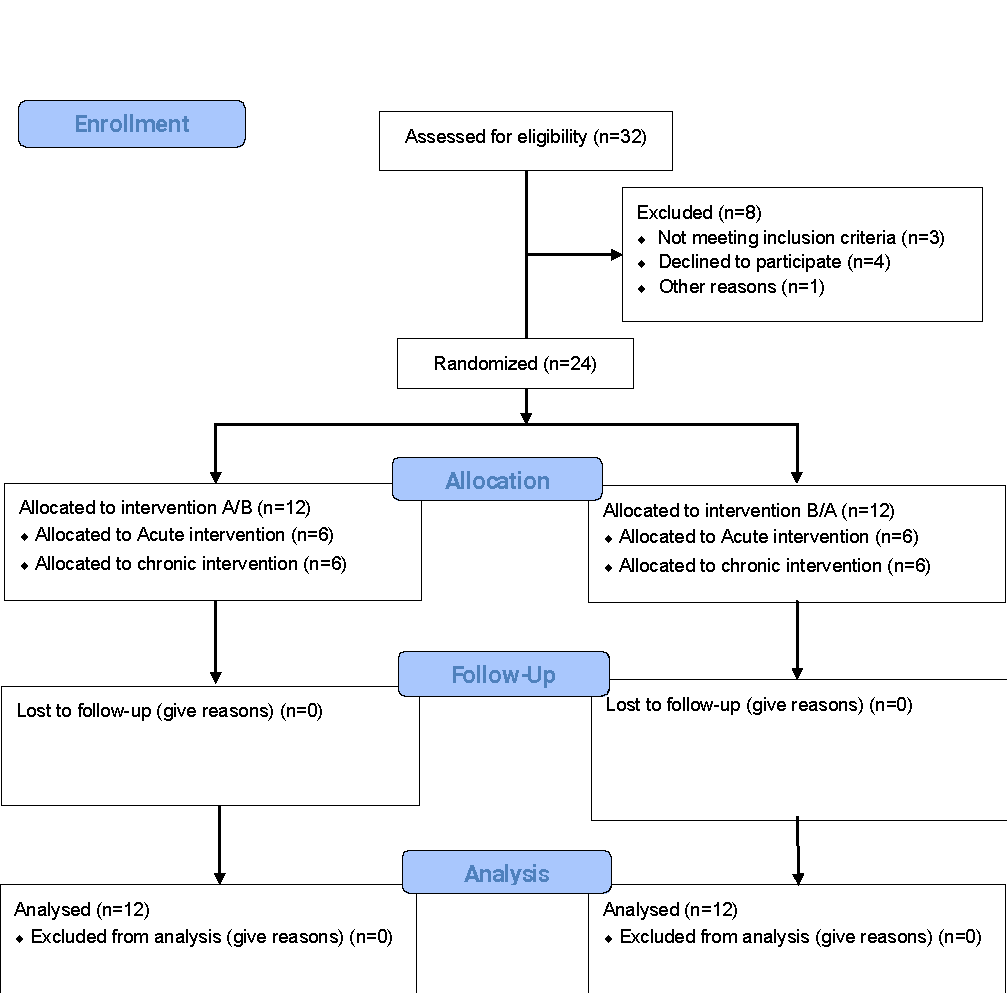


**Figure S1.** Consort diagram for King’s College London (KCL)  **Figure S2.** Consort diagram for Maastricht University (MU)


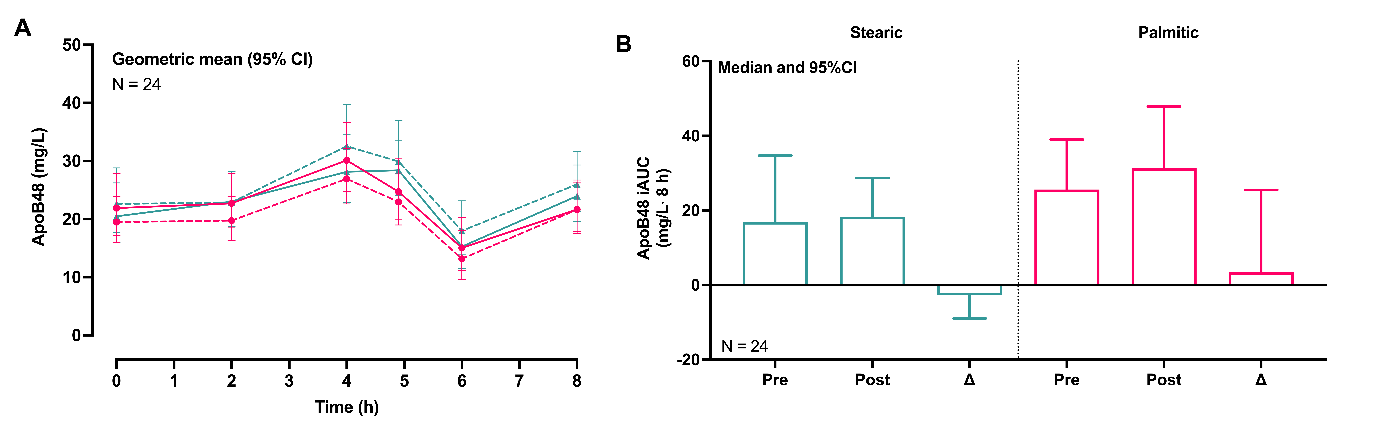


**Figure S3.** Time averaged incremental area under curve (iAUC0-8h, 95% CI) following test meals containing 50 g of 18:0-rich fat (Stearic arm) or 16:0-rich fat (Palmitic arm) pre- (baseline) and post- (endpoint) a chronic dietary intervention comprising 6 week of daily test fat consumption. Statistical analysis used linear mixed models: changes from baseline as a dependent variable; fixed effects, dietary intervention group (repeated effect), visit number (dietary intervention order), dietary intervention × visit number, center, dietary intervention x center, baseline value (covariate), dietary intervention x baseline value; and participant ID was included as a random effect. No significant differences between or within treatments were observed. Box plots represent median with interquartile ranges and whiskers represent the minimum and maximum values.


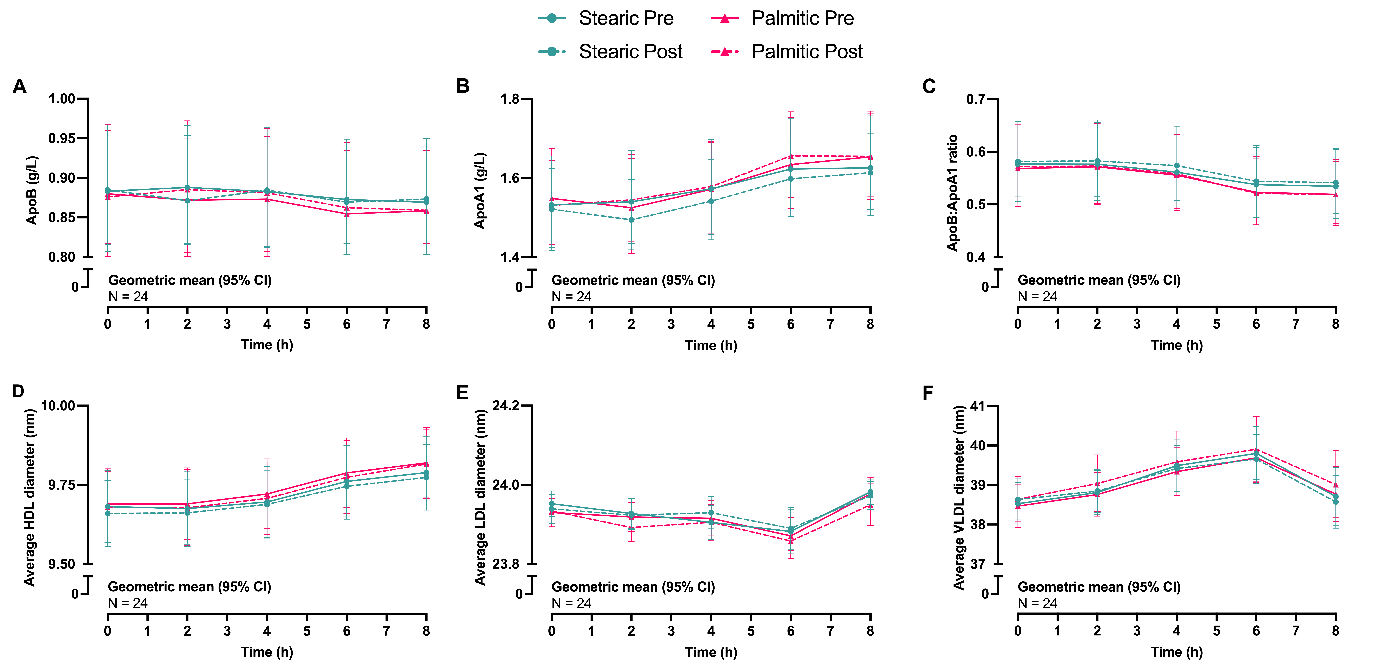


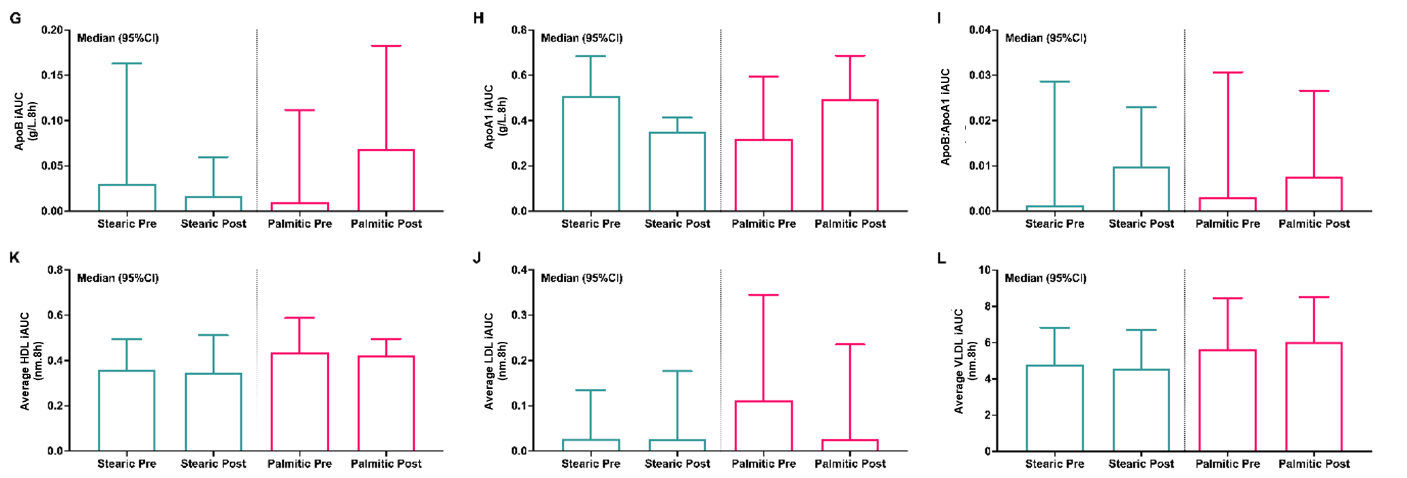


**Figure S4.** Postprandial (0-8 h) serum **A**) total ApoB concentrations (g/L), **B**) ApoA1 concentrations (g/L), **C**) ApoB:ApoA1 ratio, **D**) average HDL diameter (nm), **E**) average LDL diameter (nm) and **F**) average VLDL diameter (nm). Postprandial incremental area under curve (iAUC0-8h) for serum **G**) total ApoB concentrations, H) ApoA1 concentrations, I) ApoB:ApoA1 ratio, **J**) average HDL diameter, **K**) average LDL diameter and **L**) average VLDL diameter in following 18:0-rich fat and 16:0-rich fat test meals pre- and post- 6 week of daily test fat consumption. Statistical analysis used linear mixed models. **A-F**: changes from baseline as a dependent variable; fixed effects included dietary intervention group (repeated effect), visit number (dietary intervention order), dietary intervention × visit number, timepoint (repeated effect), dietary intervention x timepoint, dietary intervention x timepoint x visit, baseline value (covariate), dietary intervention x baseline value, center, dietary intervention x center; and participant ID was included as a random effect. **G-L**: iAUC0-8h as dependent variables; fixed effects included dietary intervention group (repeated effect), visit number (dietary intervention order), dietary intervention × visit number, center, dietary intervention x center, baseline value (covariate), dietary intervention x baseline value; and participant ID was included as a random effect. No significant differences were observed either between dietary interventions or within arms. Box plots represent median with interquartile ranges and whiskers represent the minimum and maximum values.


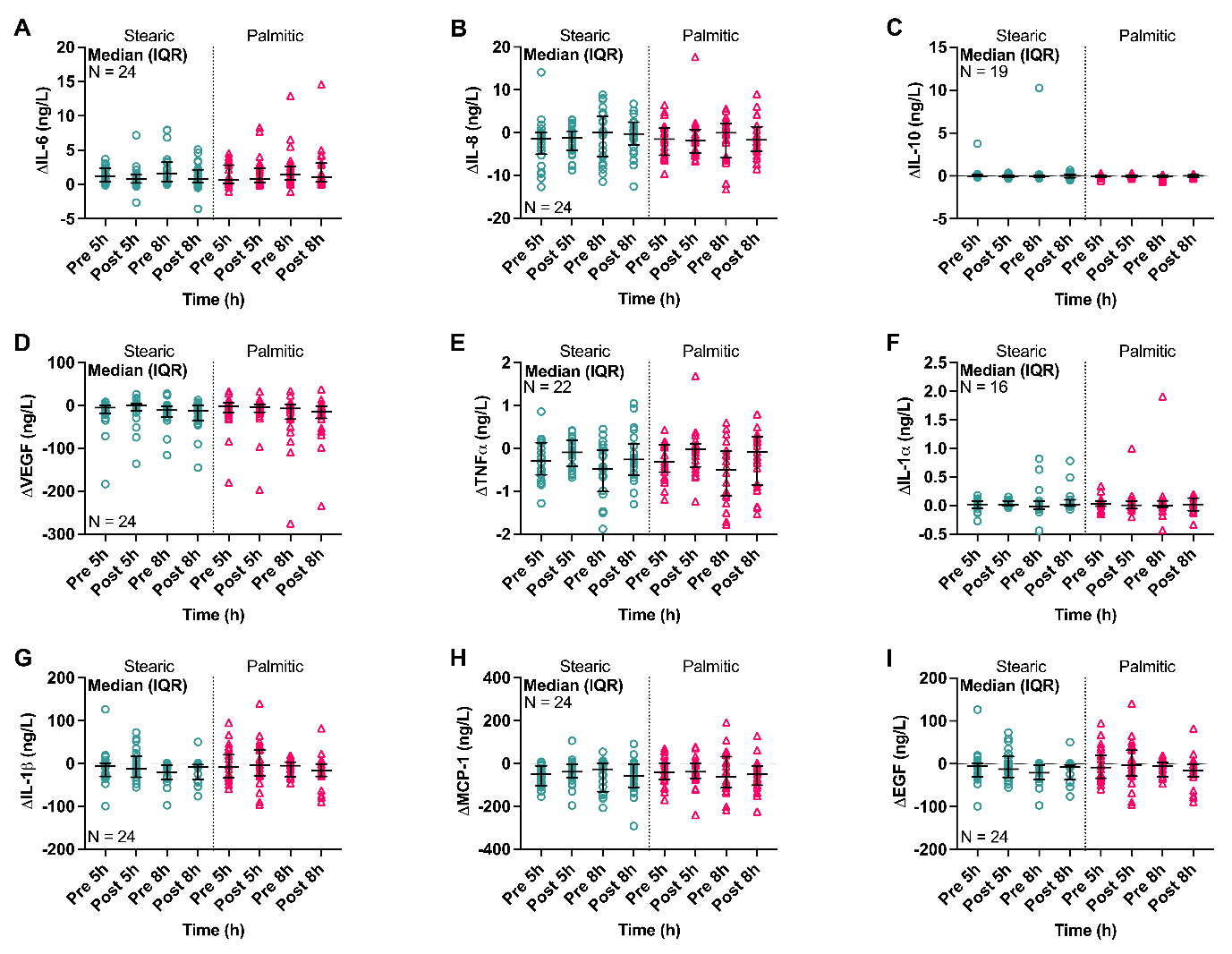


**Figure S5.** Postprandial change in serum concentrations of **A**) IL-6 **B**) IL-8 **C**) IL-10 **D**) VEGF **E**) TNF-α **F**) IL-1α **G**) IL-1β **H**) MCP1 and **I**) EGF at 5h and 8h following 18:0-rich fat and 16:0-rich fat test meals at baseline (pre-intervention) and endpoint (post-intervention) following 6 weeks of daily test fat consumption. Statistical analysis used linear mixed models: changes from baseline as a dependent variable; fixed effects included dietary intervention group (repeated effect), visit number (dietary intervention order), dietary intervention × visit number, center, dietary intervention x center, baseline value (covariate), dietary intervention x baseline value; and participant ID was included as a random effect. Significant differences between dietary interventions for changes in 18:0 and 16:0 proportions in total plasma were noted at fasting and postprandially. No significant differences were observed either between treatments or within arms.


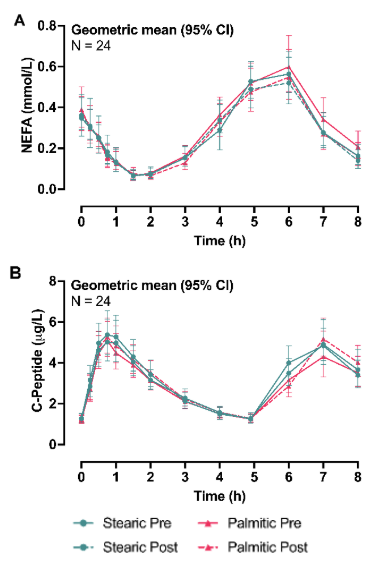


**Figure S6.** Postprandial change in **A**) serum NEFA, and **B**) serum C-peptide over 8h following 18:0-rich fat and 16:0-rich fat test meals at baseline (pre-intervention) and endpoint (post-intervention) following 6 weeks of daily test fat consumption (Stearic or Palmitic, n=24). Statistical analysis used linear mixed models: changes from baseline as a dependent variable; fixed effects included dietary intervention group (repeated effect), visit number (dietary intervention order), dietary intervention × visit number, timepoint (repeated effect), dietary intervention x timepoint, dietary intervention x timepoint x visit, baseline value (covariate), dietary intervention x baseline value, center, dietary intervention x center; and participant ID was included as a random effect. No significant differences between treatments or within arms (baseline compared to 6-wk endpoint) were observed.


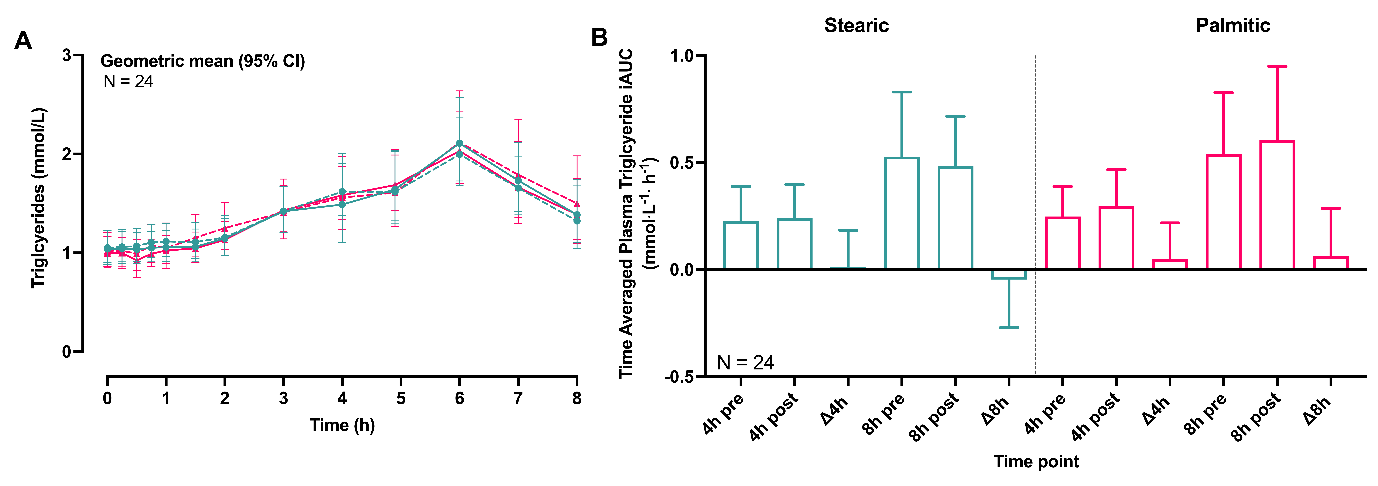
 **Figure S7.** Plasma TAG (mmol/L) as time averaged incremental area under curve (iAUC0-4h and iAUC0-8h; 95% CI) following test meals containing 50 g of 18:0-rich fat (Stearic arm) or 16:0-rich fat (Palmitic arm) pre-intervention (baseline) and post-intervention (endpoint) comprising 6 wk daily test fat consumption (Stearic or Palmitic; n=24). Statistical analysis used linear mixed models: iAUC as a dependent variable; fixed effects included dietary intervention group (repeated effect), visit number (dietary intervention order), dietary intervention × visit number, center, dietary intervention x center, baseline value (covariate), dietary intervention x baseline value; and participant ID was included as a random effect. No significant differences between or within treatments were observed.

**Table S1.** Nutrient composition of muffins used as snacks in the chronic dietary intervention and as high-fat test meals in the postprandial test days

|  | Chronic dietary intervention | | | Postprandial test days | |
| --- | --- | --- | --- | --- | --- |
|  | Chilli/garlic & parsley,  per 100 g (per muffin) | Cheese,  per 100 g (per muffin) | Sweet,  per 100 g (per muffin) | First high-fat test meal (2 x sweet muffins, plus milkshake) | Second high-fat test meal (3 x cheese muffins) |
| One muffin, g | 33.1 | 34.9 | 24.2 | 171 per portion (muffin) | 169.8 per portion |
| Energy, kcal | 245 (81) | 244 (85) | 331 (80) | 897 | 550 |
| Energy, kJ | 1029 (341) | 1025 (357) | 1390 (336) | 3753 | 2301 |
| Fat, g | 10 (3.3) | 9.2 (3.2) | 13 (3.2) | 50 | 30 |
| Carbohydrate, g | 31 (10) | 34 (12) | 45 (11) | 88 | 56 |
| Sugar, g | 0.5 (0) | 2.3 (0.8) | 25 (6.1) | 29.2 | 4.4 |
| Protein, g | 6 (2) | 5.9 (2) | 8 (1.9) | 16 | 14 |
| Salt, g | 1.2 (0.4) | 1.3 (0.5) | 0.6 (0.2) | 1.1 | 0.7 |

**Chronic dietary intervention**. Chilli/garlic and parsley muffins had the following ingredients: white self-raising wheat flour (43.2%), water (36.2%), Stearic or Palmitic hardstock fat (9.4%), eggs (8.6%), dried egg white (<1%), salt (0.5%), baking powder (<0.5%) and chili powder (<1%). Cheese muffins contained: white self-raising wheat flour (41%), water (32%), Stearic or Palmitic hardstock fat (8.2%), cheese powder (5.7%), skimmed milk (4.1%), salt (0.5%), and baking powder (<0.5%). Ingredients of sweet muffins included white self-raising wheat flour (26.8%), sugar (24.6%), eggs (17.9%), Stearic or Palmitic hardstock fat (12.7%), skimmed milk (8.3%), water (4.1%), dried egg white (3.9%), baking powder (0.6%) and flavour essence (~1%).

**Postprandial test days**. First high-fat test meal: white self-raising wheat flour (17%), sugar (17%), Stearic or Palmitic hardstock fat (30%), skimmed milk (23%), cornstarch (6%), dried egg white (2%), baking powder (1%), vanilla essence (2.4%), salt (0.7%). Second high-fat test meal (cheese muffins): white self-raising wheat flour (43%), water (20%), Stearic or Palmitic hardstock fat (15%), cheese powder (6.6%), egg white liquid (13%), egg white powder (1.3%), and baking powder (1.3%).

**Table S2.** Changes in fasting lipoprotein particle concentration at baseline, endpoint and changes following 6-weeks dietary intervention (Stearic or Palmitic)

|  |  | **Stearic (nmol/L)** |  |  | **Palmitic (nmol/L)** |  | **Mean Difference^1^**  **Δ Stearic – Δ Palmitic**  **Geomean (95% CI)**  **pmol/L** |
| --- | --- | --- | --- | --- | --- | --- | --- |
|  | **Baseline** | **Endpoint** | **Δ** | **Baseline** | **Endpoint** | **Δ** |  |
| **Fasting** | **Geomean (95% CI)** | **Geomean (95% CI)** | **Median (IQR)** | **Geomean (95% CI)** | **Geomean (95% CI)** | **Median (IQR)** |  |
| XL-HDL-P | 0.27 (0.20, 0.33) | 0.25 (0.20, 0.31) | -0.002 (-0.04, 0.04) | 0.27 (0.21, 0.34) | 0.27 (0.20, 0.33) | -0.01 (-0.03, 0.02) | -5.60 (-15.36, 4.26) |
| L-HDL-P | 1.75 (1.28, 2.22) | 1.65 (1.23, 2.07) | -0.09 (-0.40, 0.22) | 1.81 (1.32, 2.30) | 1.74 (1.26, 2.22) | -0.06 (-0.33, 0.20) | -4.39 (-48.97, 42.28) |
| M-HDL-P | 4.20 (3.75, 4.65) | 4.15 (3.75, 4.54) | -0.05 (-0.59, 0.48) | 4.27 (3.79, 4.74) | 4.18 (3.76, 4.61) | -0.09 (-0.92, 0.73) | 3.03 (-41.85, 50.02) |
| S-HDL-P | 10.31 (9.75, 10.86) | 10.40 (9.90, 10.90) | -0.01 (-0.73, 0.71) | 10.40 (9.89, 10.90) | 10.32 (9.79, 10.85) | -0.04 (-1.66, 1.59) | 14.60 (-21.26, 51.78) |
| L-LDL-P | 0.81 (0.73, 0.88) | 0.80 (0.74, 0.86) | 0.01 (-0.09, 0.10) | 0.80 (0.73, 0.87) | 0.80 (0.73, 0.87) | -0.003 (-0.09, 0.09) | 1.66 (-23.07, 27.01) |
| M-LDL-P | 0.31 (0.28, 0.35) | 0.31 (0.28, 0.35) | 0.01 (-0.06, 0.07) | 0.32 (0.28, 0.36) | 0.31 (0.28, 0.35) | -0.003 (-0.05, 0.041) | 3.42 (-11.61, 18.68) |
| S-LDL-P | 0.18 (0.17, 0.20) | 0.18 (0.17, 0.20) | 0.01 (-0.03, 0.03) | 0.18 (0.17, 0.20) | 0.18 (0.17, 0.20) | 0.001 (-0.02, 0.02) | 2.02 (-6.52, 10.64) |
| XXL-VLDL/chylo-P | 0.001 (0.0004, 0.0011) | 0.001 (0.0004, 0.0012) | -0.00001 (-0.0004, 0.0004) | 0.001 (0.0003 0.0011) | 0.001 (0.0004, 0.0012) | -0.000001(-0.001, 0.001) | 0.13 (-0.51, 0.41)^2^ |
| XL-VLDL-P | 0.003 (0.002, 0.004) | 0.003 (0.002, 0.004) | 0.00002 (-0.001, 0.001) | 0.003 (0.002, 0.004) | 0.003 (0.002, 0.004) | 0.0001 (-0.001, 0.002) | -0.41 (-1.28, 0.73)^2^ |
| L-VLDL-P | 0.01 (0.008, 0.013) | 0.010 (0.008, 0.013) | -0.0001 (-0.004, 0.003) | 0.010 (0.007, 0.012) | 0.01 (0.008, 0.013) | 0.0004 (-0.003, 0.004) | -1.41 (-3.81, 2.61)^2^ |
| M-VLDL-P | 0.037 (0.031, 0.043) | 0.037 (0.032, 0.043) | 0.002 (-0.009, 0.012) | 0.037 (0.031, 0.042) | 0.037 (0.031, 0.042) | -0.001 (-0.008, 0.005) | 0.04 (-2.97, 3.06) |
| S-VLDL-P | 0.038 (0.033, 0.043) | 0.038 (0.033, 0.043) | 0.001 (-0.006, 0.008) | 0.038 (0.033, 0.043) | 0.038 (0.032, 0.043) | 0.0003 (-0.006, 0.007) | 0.50 (-2.13, 3.14) |
| XS-VLDL-P | 0.051 (0.047, 0.056) | 0.05 (0.047, 0.054) | 0.0001 (-0.0051, 0.0054) | 0.05 (0.046, 0.055) | 0.050 (0.045, 0.054) | -0.0004 (-0.01, 0.01) | -0.27 (-2.95, 2.42) |

XS, extra small; S, small; M, medium; L, large; XL, extra-large; XXL, extra-extra-large; P, particle concentration; chylo, chylomicron. HDL, High-density lipoprotein; LDL, low-density lipoprotein; VLDL, very low-density lipoprotein. Δ = endpoint – baseline. Geomean; geometric mean.

N = 47 included in analysis. Statistical analysis was conducted by linear mixed models including changes from baseline as the dependent variable, intervention arm (repeated effect), visit number (dietary intervention order), intervention arm × visit number, study center, intervention arm x study center, baseline value (covariate), intervention arm x baseline value as fixed effects, and participant identifier as a random effect. There were no significant differences between treatments.

^1^Estimated marginal means from linear mixed model. ^2^Residuals of mixed model non-normal, data presented as Median (IQR) and difference in median change between treatments assessed by Wilcoxon rank sum test.

**Table S.3** Glucose, insulin, c-peptide, and non-esterified fatty acid (NEFA) postprandial peak concentrations (cmax) and incremental area under curve (iAUC) following high-fat test meals rich in 18:0 or 16:0, shown both at baseline, endpoint and changes following 6-weeks dietary intervention (Stearic or Palmitic)

|  |  | **Stearic** |  |  | **Palmitic** |  | **Mean difference^1^**  **Δ Stearic – Δ Palmitic** |
| --- | --- | --- | --- | --- | --- | --- | --- |
|  | Baseline | **Endpoint** | **Δ** | **Baseline** | **Endpoint** | **Δ** |  |
|  | **Geomean (95CI)** | **Geomean (95CI)** | **Median (IQR)** | **Geomean (95CI)** | **Geomean (95CI)** | **Median (IQR)** |  |
| **Glucose (mmol/L)** |  |  |  |  |  |  |  |
| ***Cmax*** | 8.09 (7.98, 8.2) | 8.47 (8.37, 8.57) | 0.25 (-1.30, 2.30) | 8.48 (8.38, 8.58) | 8.13 (8.00, 8.27) | -0.50 (-2.20, 1.50) | 0.9 (-0.25, 1.52) ^2^ |
| ***iAUC 2h*** | 1.13 (0.72, 1.66) | 1.22 (0.80, 1.74) | 0.06 (-0.60, 0.53) | 1.13 (0.75, 1.61) | 1.32 (0.9, 1.82) | 0.20 (-0.44, 0.70) | -0.06 (-0.24, 0.16) |
| ***iAUC 5h*** | 1.32 (0.88, 1.87) | 1.41 (0.94, 2.00) | 0.15 (-0.64, 0.47) | 1.48 (0.99, 2.08) | 1.73 (1.18, 2.41) | -0.01 (-0.46, 0.89) | 0.00 (-0.23, 0.3) |
| ***iAUC 8h*** | 4.76 (3.56, 6.28) | 5.04 (3.67, 6.82) | 0.02 (-0.66, 1.29) | 4.84 (3.41, 6.74) | 4.95 (3.61, 6.68) | 0.27 (-1.07 ,1.71) | 0.02 (-0.22, 0.33) |
| ***iAUC 5-8h*** | 3.35 (2.41, 4.54) | 3.51 (2.38, 5.02) | 0.44 (-0.94, 1.45) | 3.39 (2.28, 4.87) | 3.21 (2.22, 4.51) | 0.02 (-0.97, 1.69) | 0.08 (-0.23, 0.5) |
| **Insulin (mIU/L)** |  |  |  |  |  |  |  |
| ***Cmax*** | 24.02 (21.22, 27.16) | 42.09 (37.57, 47.14) | 1.21 (-67.7, 90.5) | 19.93 (17.72, 22.39) | 17.08 (15.06, 19.35) | -0.02 (-88.0, 84.8) | 2.03 (-9.66, 59.03) ^2^ |
| ***iAUC 2h*** | 68.4 (50.9-92.0) | 78.0 (61.1, 99.6) | 7.8 (-14.5 ,17.7) | 64.0 (49.1, 83.4) | 73.3 (57.6, 93.3) | 10.19 (-5.0, 24.8) | 0.00 (-0.18, 0.21) |
| ***iAUC 5h*** | 84.2 (62.5, 113.3) | 90.7 (70.0, 117.4) | 12.29 (-24.3, 22.4) | 76.8 (57.8, 101.9) | 90.1 (70.08, 115.9) | 15.20 (-1.9, 26.2) | -0.07 (-0.24, 0.14) |
| ***iAUC 8h*** | 159.8 (120.9, 211.2) | 167.0 (128.3, 217.2) | -1.58 (-33.6, 27.1) | 139.9 (106.5, 183.8) | 165.2 (130.5, 209.0) | 13.06 (-7.2, 44.9) | -0.11 (-0.29, 0.11) |
| ***iAUC 5-8h*** | 74.3 (56.0, 98.6) | 73.9 (54.3, 100.5) | -1.74 (-33.0, 14.2) | 57.8 (40.7, 82.0) | 74.2 (58.0, 94.8) | 11.05 (-5.7, 30.8) | -0.22 (-0.45, 0.1) |
| **C-Peptide (ug/L)** |  |  |  |  |  |  |  |
| ***Cmax*** | 6.13 (5.92, 6.35) | 6.20 (5.98, 6.42) | -0.31 (-2.24, 5.81) | 5.94 (5.76, 6.12) | 5.88 (5.72, 6.04) | -0.20 (-3.34, 2.46) | 0.19 (-2.00, 2.48) ^2^ |
| ***iAUC 2h*** | 5.37 (4.29, 6.67) | 5.97 (4.89, 7.26) | 0.23 (-0.62, 1.33) | 5.21 (4.22, 6.38) | 5.61 (4.60, 6.79) | 0.60 (-0.32, 1.21) | 0.03 (-0.09, 0.17) |
| ***iAUC 5h*** | 7.86 (6.25, 9.82) | 8.21 (6.65, 10.1) | 0.54 (-0.89, 1.63) | 7.49 (6.01, 9.29) | 8.19 (6.68, 9.98) | 0.97 (-1.25, 2.28) | -0.02 (-0.15, 0.12) |
| ***iAUC 8h*** | 15.8 (13.3, 18.8) | 15.8 (12.9, 19.2) | -0.79 (-2.63, 2.45) | 14.5 (12.04, 17.43) | 15.7 (13.28, 18.46) | 1.06 (-2.25, 3.15) | -0.07 (-0.21, 0.09) |
| ***iAUC 5-8h*** | 8.02 (6.81, 9.43) | 7.63 (6.13, 9.43) | -1.12 (-2.38, 1.01) | 6.81 (5.34, 8.64) | 7.53 (6.33, 8.93) | 0.08 (-0.86, 2.65) | -0.14 (-0.32, 0.09) |
| **NEFA (mmol/L)** |  |  |  |  |  |  |  |
| ***iAUC 2h*** | 0.03 (0.01, 0.05) | 0.02 (0.00, 0.04) | 0.00 (-0.05, 0.00) | 0.01 (-0.01, 0.03) | 0.02 (0, 0.05) | 0.00 (0.00, 0.01) | 0.00 (-0.05, 0.01) ^2^ |
| ***iAUC 5h*** | 0.9 (0.09, 0.31) | 0.16 (0.08, 0.25) | 0.00 (-0.13, 0.06) | 0.15 (0.07, 0.23) | 0.17 (0.33, 0.68) | 0.00 (-0.02, 0.10) | -0.05 (-0.2, 0.13) ^2^ |
| ***iAUC 8h*** | 0.51 (0.3, 0.74) | 0.49 (0.31, 0.7) | 0.01 (-0.38, 0.25) | 0.57 (0.38, 0.8) | 0.54 (0.09, 0.25) | -0.03 (-0.17, 0.16) | 0.08 (-0.3, 0.28) ^2^ |
| ***iAUC 5-8h*** | 0.43 (0.26, 0.63) | 0.45 (0.28, 0.63) | 0.01 (-0.32, 0.25) | 0.53 (0.35, 0.74) | 0.49 (0.36, 0.75) | -0.02 (-0.18, 0.13) | 0.06 (-0.19, 0.28) ^2^ |

Δ = endpoint – baseline. N = 24 included in analysis.

Statistical analysis was conducted by linear mixed models including changes in cmax, or iAUC(0-8h) calculated by the trapezoid method, as the dependent variable; dietary intervention arm (repeated effect), visit number (dietary intervention arm order), dietary intervention × visit number, baseline value (covariate), dietary intervention x baseline value, study center, dietary intervention x study center as fixed effects; and participant ID as a random effect.

^1^Estimated marginal means from linear mixed model.

^2^Residuals of mixed model non-normal so data presented as Median (IQR) and difference in median change between treatments assessed by Wilcoxon rank sum test
